# Experimental and numerical evaluation of the mechanical behavior of alkali-activated slag concrete with recycled waste glass and dealuminated metakaolin powders

**DOI:** 10.1038/s41598-026-36359-8

**Published:** 2026-02-12

**Authors:** Marina A. Nader, Mohamed O. R. El-Hariri, Ahmed Kamar, Islam N. Fathy, Mohamed S. Saif

**Affiliations:** 1 Department of Building and Construction, Faculty of Engineering, October 6 University, Giza, Egypt; 2 Department of Civil Engineering, Faculty of Engineering at Shoubra, Benha University, Cairo, Egypt; 3 Construction and Building Engineering Department, October High Institute for Engineering & Technology, Giza, Egypt

**Keywords:** Flexural behavior, Alkali-activated slag concrete, Ground granulated blast furnace slag, Waste glass powder, Dealuminated metakaolin, Steel fibers, Finite-element analysis, Engineering, Materials science

## Abstract

This study aims to evaluate the mechanical performance of ambient-cured Alkali-Activated Slag Concrete (AASC) incorporating different coarse aggregates (dolomite and basalt), recycled waste glass powder (WGP), dealuminated metakaolin (DK), and steel fibers (SF). An experimental program was conducted to assess the mechanical properties of the investigated mixes. Microstructural characteristics were also examined using SEM-EDX analysis to support the interpretation of mechanical trends. Furthermore, finite element (FE) simulation models were developed in ABAQUS and validated against experimental results. The findings indicate that AASC incorporating 10% DK and 1% SF exhibits the highest overall mechanical performance. Based on the experimental results, a nonlinear Concrete Damaged Plasticity (CDP) model was developed and rigorously calibrated, enabling reliable FE simulation of AASC behavior. The validated model was subsequently employed in an extensive parametric study to investigate the flexural response of reinforced AASC beams, which demonstrated that the inclusion of 1% SF increased load-carrying capacity by approximately 46% while reducing mid-span deflection by about 19.7%. The results show that the optimized mixes, particularly those incorporating WGP + SF or DK + SF, achieved higher ultimate loads, reduced deflections, delayed crack initiation, and improved ductility. The proposed FE model accurately predicts load capacity and failure modes, providing a robust tool for future structural design and optimization of sustainable AASC elements utilizing industrial by-products and waste materials. The novelty of this study lies in the comprehensive investigation of recycled WGP and DK, each combined with SF in AASC, supported by integrated experimental and numerical analyses to elucidate their synergistic effects on mechanical behavior.

## Introduction

Geopolymer concrete (GPC) produced from industrial waste materials has increasingly been recognized as a viable and sustainable alternative to conventional Portland cement concrete. Incorporating locally sourced waste streams not only reduces the demand for natural raw materials but also aligns with circular-economy strategies aimed at improving resource efficiency^1–3^. Furthermore, several studies have reported that waste-based geopolymer binders can reduce embodied energy and carbon emissions by approximately 60–80% compared with ordinary Portland cement concrete, highlighting their potential to mitigate environmental impacts associated with the construction sector^4–6^. In addition to these environmental advantages, GPC also demonstrates promising mechanical performance, reinforcing its suitability for structural and infrastructure applications.

Ground Granulated Blast Furnace Slag (GGBFS), fly ash, and metakaolin are widely used in GPC. Their selection is primarily based on their high alumino-silicate content, a critical factor for the geopolymerization process^7–10^. Increasing the GGBFS content in the mixture enhances compressive, tensile, and flexural strengths, particularly at early ages, with optimal replacement levels typically ranging from 20% to 50% to achieve the best balance between workability and mechanical performance. Furthermore, slag-based GPC exhibits lower water absorption, lower permeability, and improved resistance to shrinkage and environmental degradation compared with conventional concrete^11^. A significant body of research indicates that GPC exhibits mechanical properties comparable or superior to those of conventional ordinary Portland cement concrete. Evaluations of compressive, flexural, and tensile strength, alongside elastic modulus, quantified via standardized cubic, cylindrical, and prismatic specimens^12,13^, underscore its viability for durable and sustainable infrastructure. Crushed glass, ceramic waste, crumb rubber, marble waste, silica fume, clay brick powder, waste zeolite, steel slag, and plastic waste have been used in ordinary and GPC as partial replacements for aggregates or binders^2,4,7,14–20^. Among these materials, waste glass is particularly notable due to its high amorphous silica content, making it a promising precursor for alkali-activated binders and addressing environmental concerns from the 100 million tons of waste glass generated globally each year^21^. The reactivity of fine and coarse waste glass varies with particle size and treatment conditions^22^. Incorporating waste glass powder (WGP) as a fine aggregate can improve cracking resistance, ductility, and load-carrying capacity by up to 50%^23^. Replacing 10–20% of fly ash with WGP may slightly increase compressive strength under heat curing^24^, although some studies report reduced strength even after 24-hour curing at 60 °C^25^. Replacing GGBFS with WGP has shown no improvement in ambient-cured alkali-activated mortars^26^. Despite these findings, limited research has investigated the role of WGP in ambient-cured slag-based binders, highlighting the need for further evaluation of GGBFS systems incorporating WGP.

Dealuminated metakaolin (DK) is a waste by-product generated during the aluminum extraction from calcined kaolin using sulfuric acid, where part of the aluminum is removed, producing amorphous silica with high surface area and high reactivity^27^. Its Al₂O₃/SiO₂ ratio varies according to the acid type and concentration used in the dealumination process^28^. Kaolin is abundant in Egypt and occurs in several regions, including Sinai, the Red Sea coast, and Kalabsha^29^. It is widely used in the production of cement, ceramics, rubber, paints, plastics, porcelain, and pigments^30^. The valorization of DK addresses environmental concerns associated with the approximately 80,000 tonnes generated annually^31^. Incorporating DK in GPC or cementitious systems improves compressive strength, tensile strength, and elastic modulus, particularly at elevated temperatures, acting as a nucleating agent that densifies the matrix and enhances microstructure^32^. Optimal partial replacement of cement with DK (10–15%) has been reported to increase mechanical performance, although higher ratios (15–30%) may reduce compressive strength and flowability^33–35^. Recent studies have focused on DK utilization in geopolymer development due to its amorphous silica content, high surface area, and reactivity^32,36,37^.

Several studies have reported that the incorporation of fibers and different types of structures can improve the mechanical properties of concrete^38^. Specifically, Steel fibers (SF) addition in GPC significantly enhances mechanical properties and durability, particularly at optimal dosages. Key benefits include increased compressive, splitting tensile, and flexural strengths, improved crack resistance, and enhanced toughness. Typically, SF content up to 1–2% by volume is effective; exceeding this may reduce workability without further strength gains^39–41^. Combining 1% SF with 2% nano-silica has been reported to maximize mechanical performance^42^. SF also improve elastic modulus and ductility, reducing brittleness and increasing energy absorption, which is crucial for structural applications^43,44^. Despite these advantages, studies on the durability of steel fiber-reinforced GPC remain limited^45^. This study aims to address these gaps by examining the effects of specimens with and without 1% SF.

GPC beams incorporating recycled aggregates or other waste materials generally exhibit flexural strength and load capacity that are similar to or slightly lower than those of conventional concrete^46^. However, their performance can be significantly improved through optimal material selection and mix design. Notably, beams with recycled materials often display higher ductility and greater deflection at peak loads, with failure modes shifting from brittle to more ductile as the proportion of waste increases^47,48^. When carefully optimized using specific types of waste, eco-friendly geopolymer beams can match or even exceed the flexural performance and ductility of conventional beams. In addition to mechanical advantages, these sustainable alternatives offer enhanced durability and environmental benefits, making them promising candidates for future construction applications.

Numerical and analytical models, as well as existing design codes, can generally predict the flexural capacity of GPC beams with waste materials with reasonable accuracy, although safety margins may be lower than for traditional concrete^49,50^. Despite considerable research on GPC, it remains a relatively new field compared to OPC. Challenges remain in mix design standardization, activator safety, and long-term performance. This highlights the need for further studies on GPC with different recycled waste materials to optimize formulations and support widespread adoption in the construction industry.

There is limited research on the effects of recycled binders (WGP or DK) with SF on the mechanical behavior of Alkali-Activated Slag Concrete (AASC) cured at ambient temperature. Most previous studies have focused on heat-cured GPC, which increases energy consumption. Ambient curing, in contrast, offers a more sustainable approach but often struggles to achieve high early strength^51^. The present study is designed as a systematic extension of the authors’ earlier experimental investigation on AASC^52^, which primarily focused on mixture optimization and the evaluation of fundamental mechanical and durability properties. That previous work examined the effects of incorporating WGP or DK, each in combination with SF, on compressive, splitting tensile, and flexural strengths, as well as on durability aspects such as density, water absorption, sorptivity, and porosity, leading to the identification of optimal mix compositions under ambient curing conditions. Building upon this established experimental baseline, the current study moves beyond basic strength assessment to address aspects that were not previously explored. Specifically, it provides a detailed characterization of the full stress–strain response, supported by SEM-EDX microstructural analysis to elucidate damage mechanisms and material evolution. Furthermore, a nonlinear Concrete Damaged Plasticity (CDP) constitutive model is developed and rigorously calibrated using experimental data, enabling reliable numerical simulation of AASC behavior. The validated model is subsequently employed in an extensive parametric numerical study to investigate the flexural response of reinforced AASC beams, thereby extending the investigation from material-level behavior to structural-scale performance. Collectively, these elements clearly differentiate the present study from prior works, highlighting its novelty and providing a holistic approach for the design and optimization of sustainable AASC mixes incorporating industrial by-products and waste materials.

## Experimental program

### Materials

**Fig. 1:**
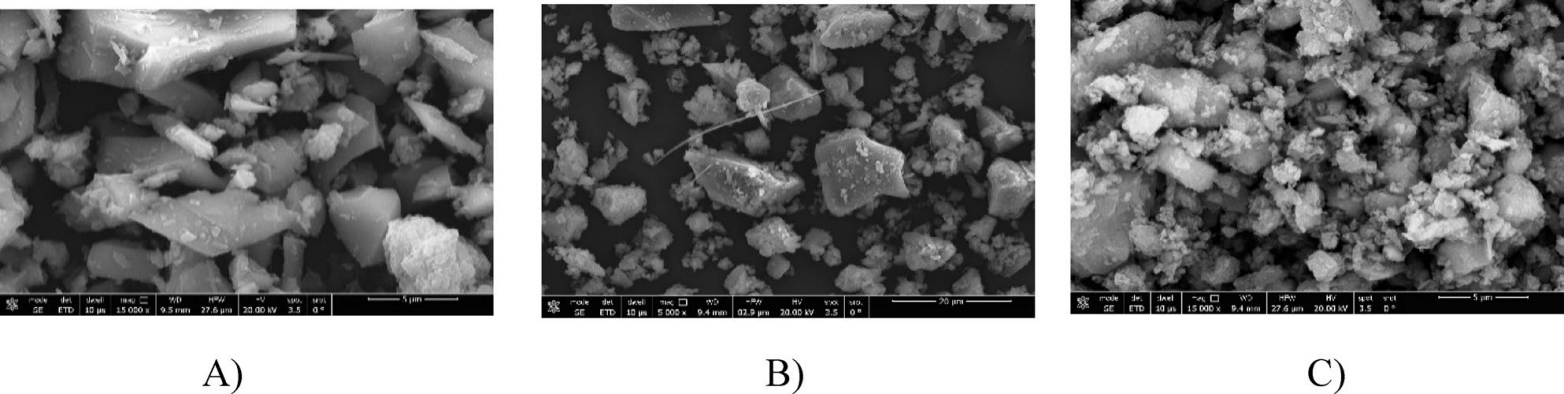
SEM images of the used materials: (**A**) GGBFS, (**B**) WGP, and (**C**) DK.

The study utilized three aluminosilicate materials: Ground Granulated Blast Furnace Slag (GGBFS), Waste Glass Powder (WGP), and Dealuminated Metakaolin (DK). GGBFS, obtained from an iron plant, has a specific gravity of 2.8 and a surface area of 4088 cm²/g. WGP has a specific gravity of 2.58 and a higher surface area of 5728 cm²/g, while DK, a by-product of aluminum sulfate production, has a specific gravity of 2.59 and a surface area of 90.5 m²/g. The chemical compositions, analyzed by XRF, are presented in Table 1. These values were taken from the authors’ previous study^52^, using the same source materials. The morphology and particle characteristics were examined using SEM, with the results shown in Fig. 1. GGBFS consists of moderately sized, irregular, angular particles with rough and heterogeneous surfaces, exhibiting numerous edges, crevices, and surface features that provide abundant reactive sites for gradual geopolymerization, and its balanced composition of CaO, SiO_2_, and Al₂O₃ supports progressive gel formation. WGP is composed of smaller, smooth, plate-like particles with sharp edges and elongated needle-like structures, displaying relatively clean surfaces that dissolve rapidly in alkaline environments, while its largely amorphous structure and high silica content further enhance its reactivity. DK contains very fine, layered particles arranged in a complex three-dimensional structure with thin sheets, plate-like crystals, and rod-shaped variations, making it highly reactive, while its richness in silica and alumina provides reactive aluminum necessary for forming strong aluminosilicate networks, thus making it an excellent source for geopolymer formation. These morphological details provide a more complete definition of the particle characteristics shown in Fig. 1, making the figure self-explanatory and highlighting differences in surface features and structure among the materials.

Natural siliceous sand of medium grading, conforming to limitations of the Egyptian Code of Practice (2018)^53^, was used, with a specific gravity of 2.5 g/cm³, water absorption of 1.53%, and fineness modulus of 2.6. Crushed dolomite and basalt from a local quarry (maximum size 20 mm) served as coarse aggregates. All aggregates were used in saturated surface dry condition. Sample preparation and testing followed BS 812, Part 110 (1990)^54^. The properties of coarse aggregates, summarized in Table 2, were taken from the authors’ previous study^52^.

The study employed undulated round SF with a length of 25 mm and a diameter of 0.8 mm. The fibers had a density of 7.86 g/cm^3^ and a tensile strength of 1100 N/mm².

The alkaline activator solution (AAS) consisted of a liquid mixture of Na_2_SiO_3_ and NaOH solution, with NaOH at a concentration of 14 M. NaOH was supplied in solid pellet form with 98–99% purity, and Na₂SiO₃ was provided in liquid form, having specific gravities of 2.13 and 1.4, respectively. The AAS was prepared with a Na_2_SiO_3_: NaOH ratio of 2.5:1 and allowed to rest at room temperature for 24 h until it reached a lukewarm temperature before use.

A high-range, water-reducing, polycarboxylic ether-based superplasticizer was incorporated into the geopolymer mixture to enhance the workability of fresh GPC. The superplasticizer had a density of 1.1 kg/L, a chlorine content below 0.1%, and an alkaline content below 3%, in compliance with EN 480 − 10 and EN 480 − 12.

**Table 1:** Chemical composition of the used materials: GGBS, WGP, and DK.

Constituents (%)	GGBFS	WGP	DK
SiO_2_	35.40	76.84	68.1
Al_2_O_3_	17.40	2.12	10.22
Fe_2_O_3_	1.40	0.5	0.65
CaO	36.87	5.23	0.59
MgO	7.83	3.29	0.1
MnO	0.18	0.03	0.01
Na_2_O	0.34	6.39	13.5
K_2_O	-	0.34	0.09
TiO_2_	0.38	0.16	2.74
SO_3_	0.2	1.62	2.55
L.O.I	-	2.89	1.15

**Table 2:** Properties of coarse aggregates.

Property	Dolomite	Basalt
Specific gravity	2.65	2.81
Unit weight (tons/m^3^)	1.56	2.85
Absorption percentage	1.41%	0.52%
Clay and other fine materials (%)	0.53%	0.32%
Abrasion value (loss angles) (%)	26.5%	14.6%
Elongation index (%)	4.12%	3.02%
Crushing value (ACV) %	23.19%	11.14%

### Concrete mixes and proportions

Based on previous studies showing positive effects of incorporating WGP^55–57^ and DK^58,59^ at similar proportions, the selected percentages were verified through preliminary laboratory trials and applied in the mixes shown in Table 3. These mixes, chosen from a prior experimental study by the authors, combined the optimal percentages with crushed coarse aggregates (dolomite and basalt) to achieve the best mechanical performance^52^. In that study, a total of fourteen different mixtures were designed to systematically examine the effects of coarse aggregate type (dolomite, granite, and basalt), WGP content (5–20% as partial sand replacement), DK content (5–15% by weight of GGBFS), and SF volume fraction (0.5–2.0%). The results demonstrated that mixtures incorporating basalt aggregate with 1% SF and either 15% WGP or 10% DK achieved the most favorable overall performance. In particular, the mixture containing 10% DK and 1% SF consistently outperformed its WGP-based counterpart in terms of strength developmentand microstructural refinement. Accordingly, these mixtures were identified as optimal formulations and were selected for the extended investigation presented in this study.

The coding of mixes is summarized in Table 3. The control mix is AASC with crushed dolomite aggregate (MD), while mix (MB) is AASC with basalt aggregate. The third mix, containing crushed basalt aggregate and 15% WGP, is referred to as (MB-15G), and when 1% SF is added, it is identified as (MB-15G-1 S). Finally, when 10% DK is added instead of WGP, the mix is called (MB-10DK-1 S).

GGBFS was used as the binder in all mixes at a constant amount of 450 kg/m^3^. Na_2_SiO_3_, NaOH, and a polycarboxylic ether-based superplasticizer were added to all mixes in the same amounts of 195 kg/m^3^, 78 kg/m^3^, and 13 L/m^3^, respectively.

**Table 3:** AASC mix proportions (Kg/m^3^).

Mix No.	Fine aggregates	Coarse aggregates		WGP	DK	SF
		D	B			
MD	630	1170	-	-	-	-
MB	630	-	1170	-	-	-
MB-15G	608	-	1123	67.5	-	-
MB-15G-1S	600	-	1105	67.5	-	79
MB-10DK-1S	606	-	1120	-	45	79

### Concrete mixing, casting, and curing

**Fig. 2:**
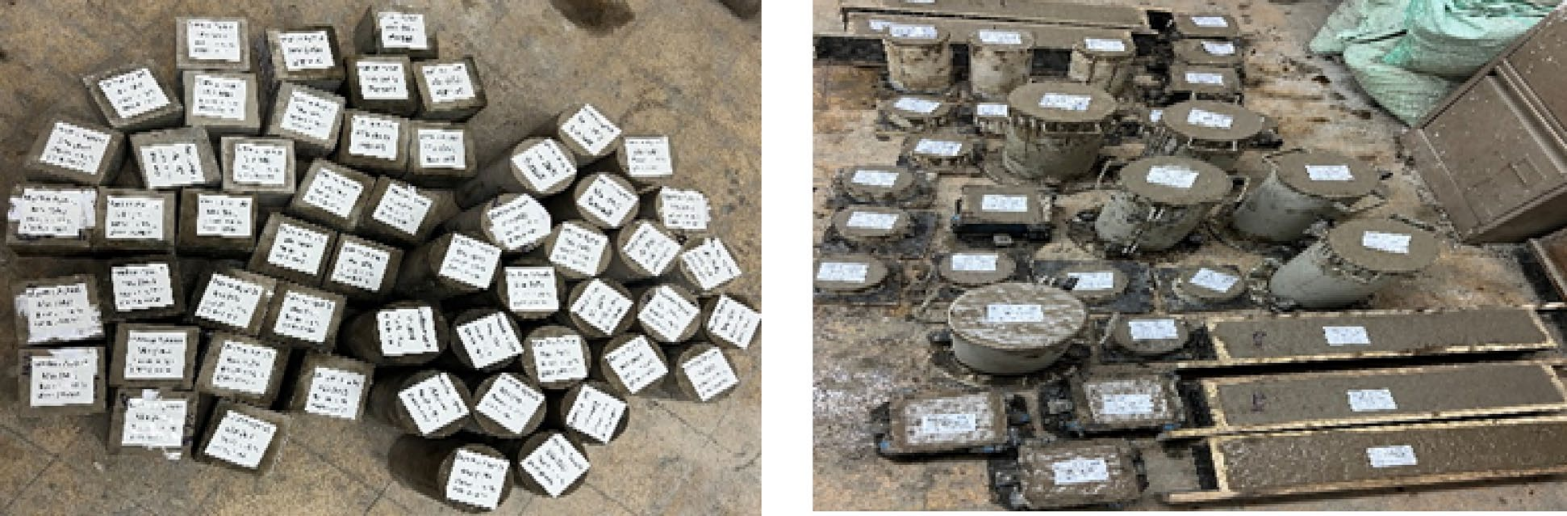
AASC specimens’ preparation.

The dry materials, such as aggregates and slag, were mixed in a concrete mixer for 3 min until thoroughly combined. For mixtures containing WGP or DK and SF, the determined amounts were added gradually in small portions at regular intervals to ensure uniform distribution in the dry mix, followed by an additional 2 min of mixing. The alkaline activator solution and superplasticizer were then slowly added to the dry materials and mixed for another 4 min until a homogeneous mixture was obtained. After uniform mixing, the fresh concrete was poured into molds as shown in Fig. 2. The sizes and numbers of all specimens, along with the corresponding test measurements and the relevant standards for each test, are listed in Table 4. After 24 h of curing at ambient temperature, the specimens were removed from molds and stored at room temperature until testing.

**Table 4:** Summary of test specimens, geometric dimensions, and corresponding standard specifications.

Test	Shape	Size (mm)	Amounts	Standard
Compressive strength test	Cube	100 × 100 × 100	30	BS EN 12390 3^60^
Splitting tensile strength test	Cylinder	100 × 200	30	ASTM C496^61^
Flexural strength test	Prism	100 × 100 × 500	15	ASTM C78^62^
Compressive elastic modulus test	Cylinder	150 × 300	15	ASTM C469^61^

## Experimental results

**Fig. 3:**
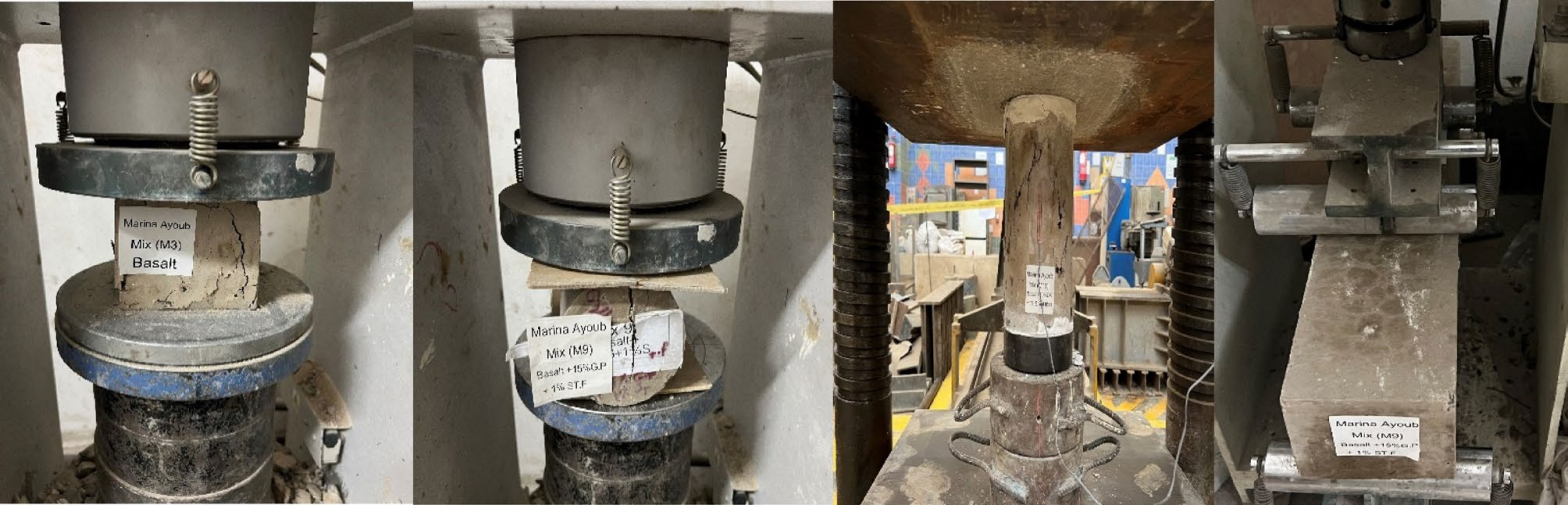
Experimental setup of mechanical property tests for AASC specimens at 28 days.

The mechanical performance of AASC mixes was evaluated through a comprehensive series of tests designed to capture both strength-related properties and the overall deformation behavior of the material. The assessment included compressive strength, splitting tensile strength, flexural capacity, and modulus of elasticity, in addition to analyzing the full stress–strain response to understand stiffness, ductility, and failure characteristics. Figure 3 presents some of the experimental tests conducted on the different AASC specimens, providing a visual representation of the testing setup and methodology. Together, these parameters offer an integrated understanding of how the various mix compositions influence the load-carrying capacity and structural performance of AASC. The following sections present and discuss the experimental findings for each mechanical property, highlighting the effects of the studied variables on the behavior of the developed GPC.

### Compressive strength

Results of the compressive strength test indicate that AASC achieves approximately 90% of its 28-day strength within the first 7 days. This rapid early strength development can be mainly associated with the high pozzolanic reactivity of the GGBFS binder used in the mixture. The relatively high calcium content is likely to react more rapidly under alkaline conditions, which may further accelerate strength gain^63^. In addition, the fine particle size of the employed GGBFS may contribute to the enhanced early-age strength through improved particle packing and reaction kinetics^64^.

**Fig. 4:**
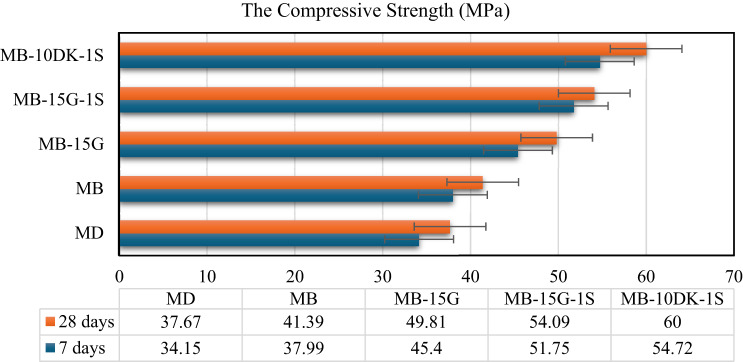
The compressive strength of AASC mixes at 7 and 28 days.

The results presented in Fig. 4 show the compressive strength of different AASC mixes. The 28-day compressive strength of mixes (MB), (MB-15G), (MB-15G-1 S), and (MB-10DK-1 S) exceeded that of the control mix (MD) by 9.88%, 32.22%, 43.59%, and 60.28%, respectively. These increases can be mainly associated with a combination of factors observed across the studied mixtures, including the lower water demand of basalt coarse aggregate in mix (MB), the pozzolanic reaction and micro-filling effects of WGP and DK in mixes (MB-15G), (MB-15G-1 S), and (MB-10DK-1 S). The use of WGP increases the Si/Al ratio of the binder system, which has been reported to improve compressive strength due to the formation of stronger Si–O–Si bonds^65^. Although WGP has low reactive alumina content, the presence of GGBFS provides sufficient calcium and alumina to support the reaction and promote the formation of C–S–H gel^66^. The positive effect of DK on AASC compressive strength can be attributed to the formation of a stable sodium-(calcium)-alumino-silicate-hydrate (N-(C)-A-S-H) gel. The silica and alumina in DK react with the alkaline solution, and the dissolution of slag particles, along with a suitable amount of Na_2_O, enhances gel formation and strength development. These results are consistent with the findings reported by Abdelalim et al.^67^. Consequently, MB-10DK-1 S achieved higher compressive strength compared to MB-15G-1 S. In addition, the presence of steel fibers (SF), which are likely to limit crack initiation and propagation in mixes containing fibers, contributes to strength improvement, as fibers increase matrix density and help control crack development under load, leading to higher compressive strength^68^. It should be noted that these interpretations are based on comparative trends observed within a limited number of mixtures.

### Splitting tensile strength

**Fig. 5:**
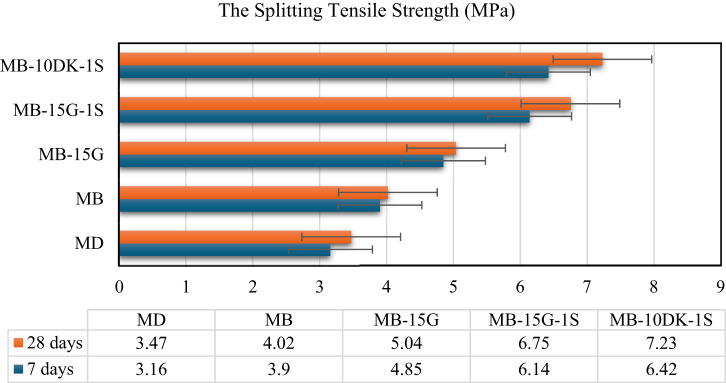
The splitting tensile strength of AASC mixes at 7 and 28 days.

The splitting tensile strengths of the control mix (MD) and mixes (MB), (MB-15G), (MB-15G-1 S), and (MB-10DK-1 S) are presented in Fig. 5. After 28 days, the splitting tensile strength of these mixes exceeded that of the control mix by 15.85%, 45.24%, 94.52%, and 108.36%, respectively. This enhancement is likely related to the same material-related trends discussed for compressive strength, including matrix densification and fiber-matrix interaction. However, these explanations represent trend-based interpretations derived from the investigated mixtures rather than statistically validated causal relationships.

### Flexural strength test

**Fig. 6:**
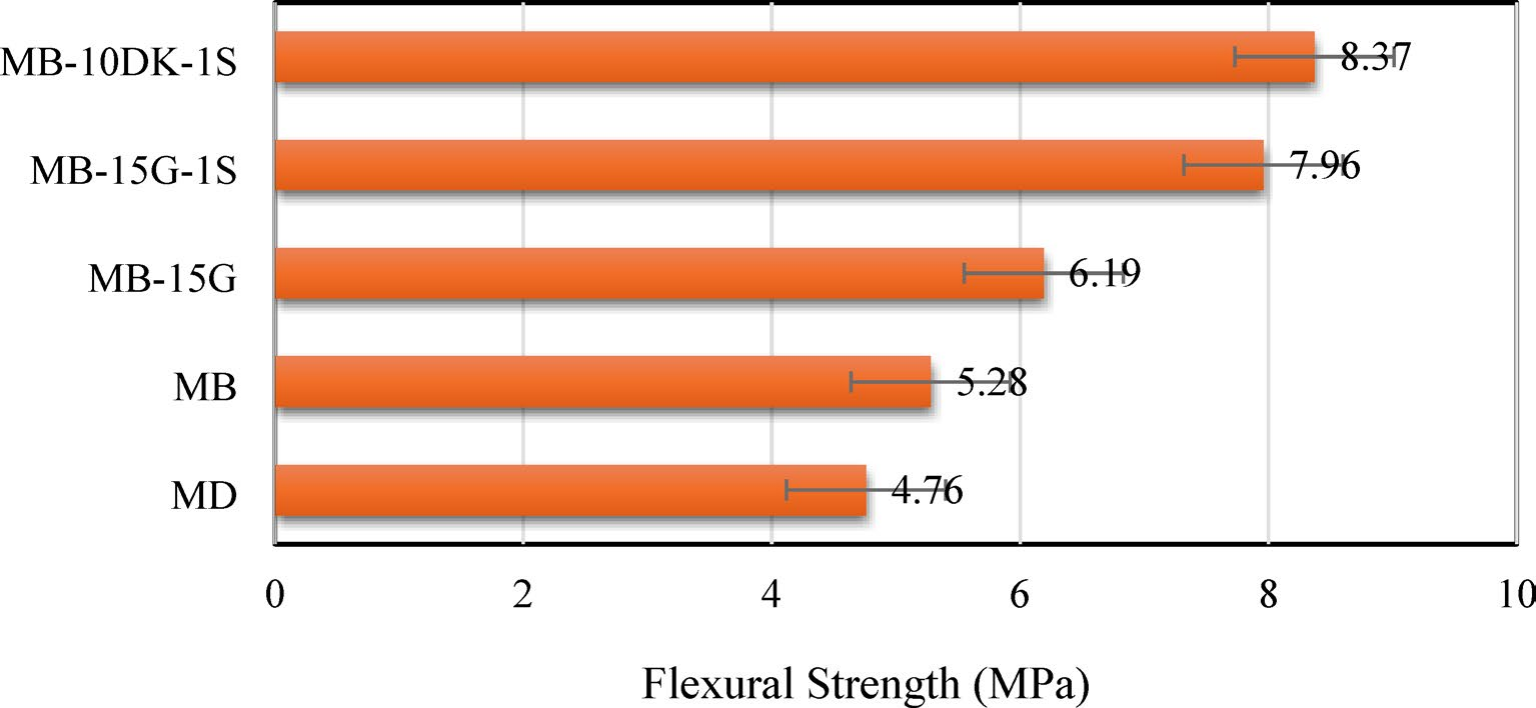
Flexural strength of AASC mixes at 28 days curing age.

The flexural strength, expressed as the modulus of rupture, was determined using two-point loading. The modulus of rupture of the control mix (MD) was 4.76 N/mm^2^, which is lower than that of mixes (MB), (MB-15G), (MB-15G-1 S), and (MB-10DK-1 S) by 9.85%, 23.1%, 40.2%, and 43.13%, respectively (Fig. 6). The observed improvement in flexural strength can be mainly associated with the higher density of basalt aggregate, the pozzolanic and filling effects of WGP and DK that may lead to a denser and more homogeneous matrix, and the inclusion of undulated round steel fibers, which are likely to enhance crack-bridging capacity and post-cracking behavior. These interpretations are inferred from observed performance trends among the studied mixes.

### Modulus of elasticity

**Fig. 7:**
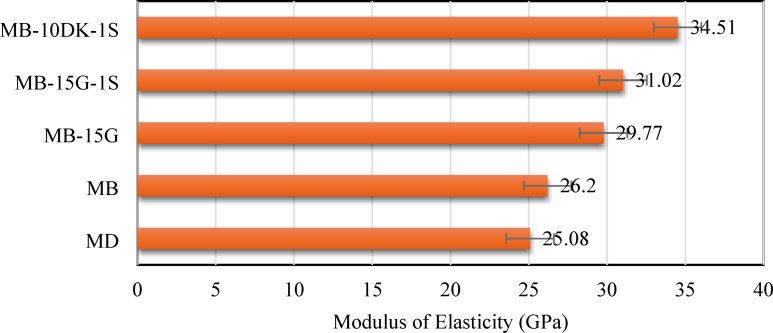
Elastic modulus of AASC mixes at 28 days curing age.

The modulus of elasticity of AASC was measured after 28 days, and the results followed a trend similar to that observed in the strength tests. The modulus of elasticity of mixes (MB), (MB-15G), (MB-15G-1 S), and (MB-10DK-1 S) exceeded that of the control mix (MD) by 4.47%, 18.7%, 23.68%, and 37.6%, respectively (Fig. 7). This improvement may be explained by the combined influence of basalt aggregate stiffness, matrix densification due to WGP and DK, and the presence of steel fibers, which are likely to enhance the overall stiffness and load-transfer efficiency of the composite. These explanations are based on comparative trends observed within the limited experimental matrix.

### Compressive stress-strain

**Fig. 8:**
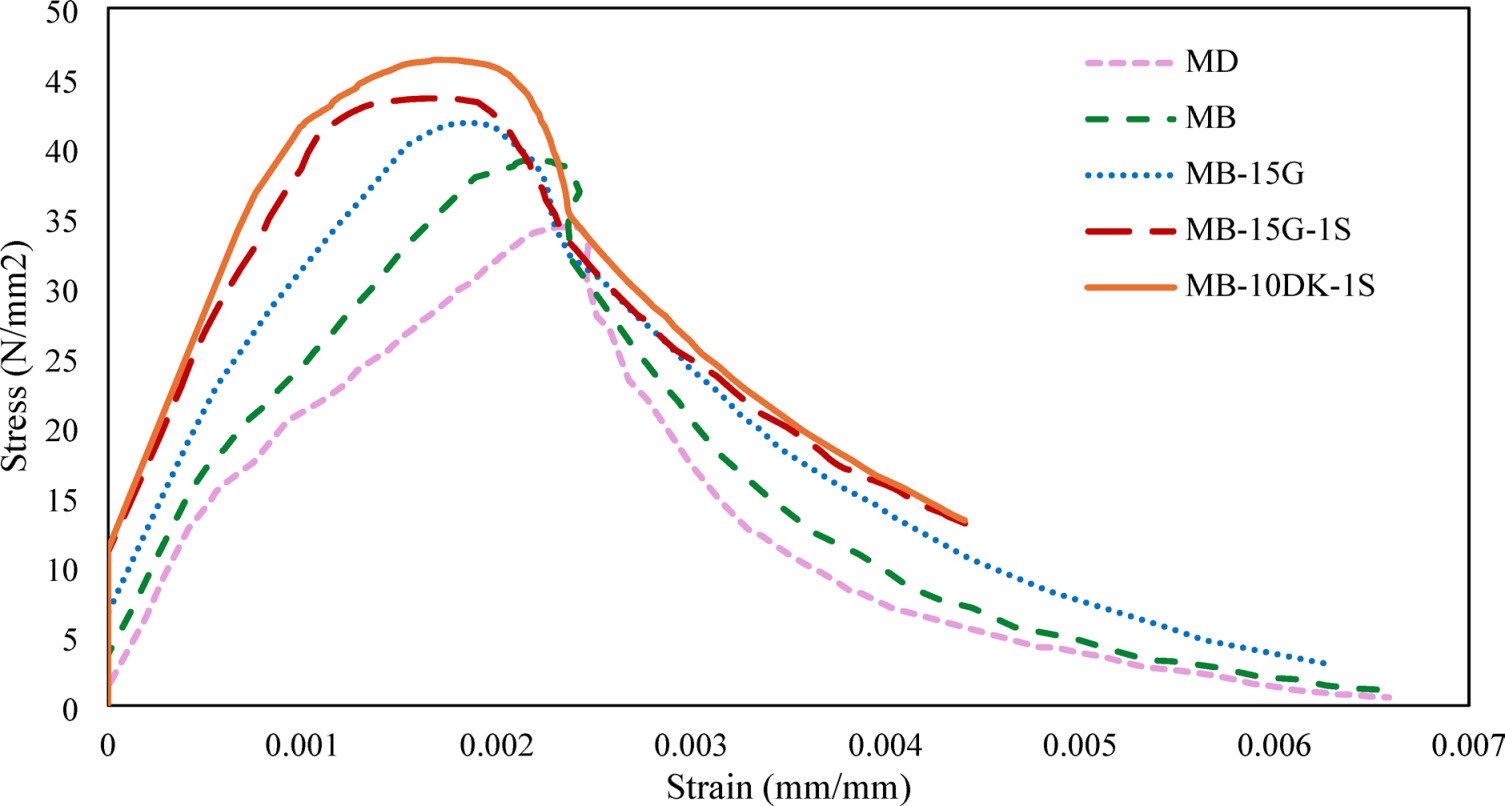
Stress-strain curve of AASC mixes at 28 days.

Compressive Stress-Strain for AASC is crucial for evaluating its mechanical behavior under axial compression. The obtained modulus of elasticity provides valuable information for structural design and numerical modeling, as well as for assessing ductility and energy absorption capacity. The inclusion of WGP or DK, and particularly 1% SF, enhanced post-peak performance, improved ductility, and increased the energy absorption capacity of the mixes compared to the control, confirming the effectiveness of these additives in modifying the stress-strain response (Fig. 8). The ascending branch of the stress–strain curves for mixtures containing SF remained almost linear up to peak stress, while mixtures without fibers showed a more rapid, brittle post-peak failure. Also, according to Abdelalim et al.^67^, a less steep descending part of the stress–strain curve corresponds to more ductile behavior. Mixtures with WGP or DK exhibited a softer post-peak decline, reflecting a moderate improvement in ductility, whereas the combination with 1% SF led to the highest toughness and delayed crack propagation. The toughness of the mixes was evaluated using a simple energy-based index, defined as the area under the compressive stress–strain curve up to a specified strain level, which clearly showed higher energy absorption for mixes containing WGP or DK, with the greatest improvement observed in mixtures with SF. This trend is further supported by the toughness values as reported in Table 5, confirming that mixtures with 1% SF achieved the highest energy absorption capacity and superior post-peak deformation compared to the control mix.

**Table 5:** Peak compressive stress, strain at peak stress, and toughness of AASC mixtures.

Specimens	Peak compressive stress (*N*/mm^2^)	Strain at peak stress	Toughness *10^− 3^ (J)
MD	34.31	0.00236	0.0867
MB	39.06	0.00211	0.1036
MB-15G	41.77	0.00184	0.1258
MB-15G-1S	43.52	0.00169	0.1278
MB-10DK-1S	46.28	0.00167	0.1339

### Microstructural analysis of AASC samples using SEM and EDX tests

**Fig. 9:**
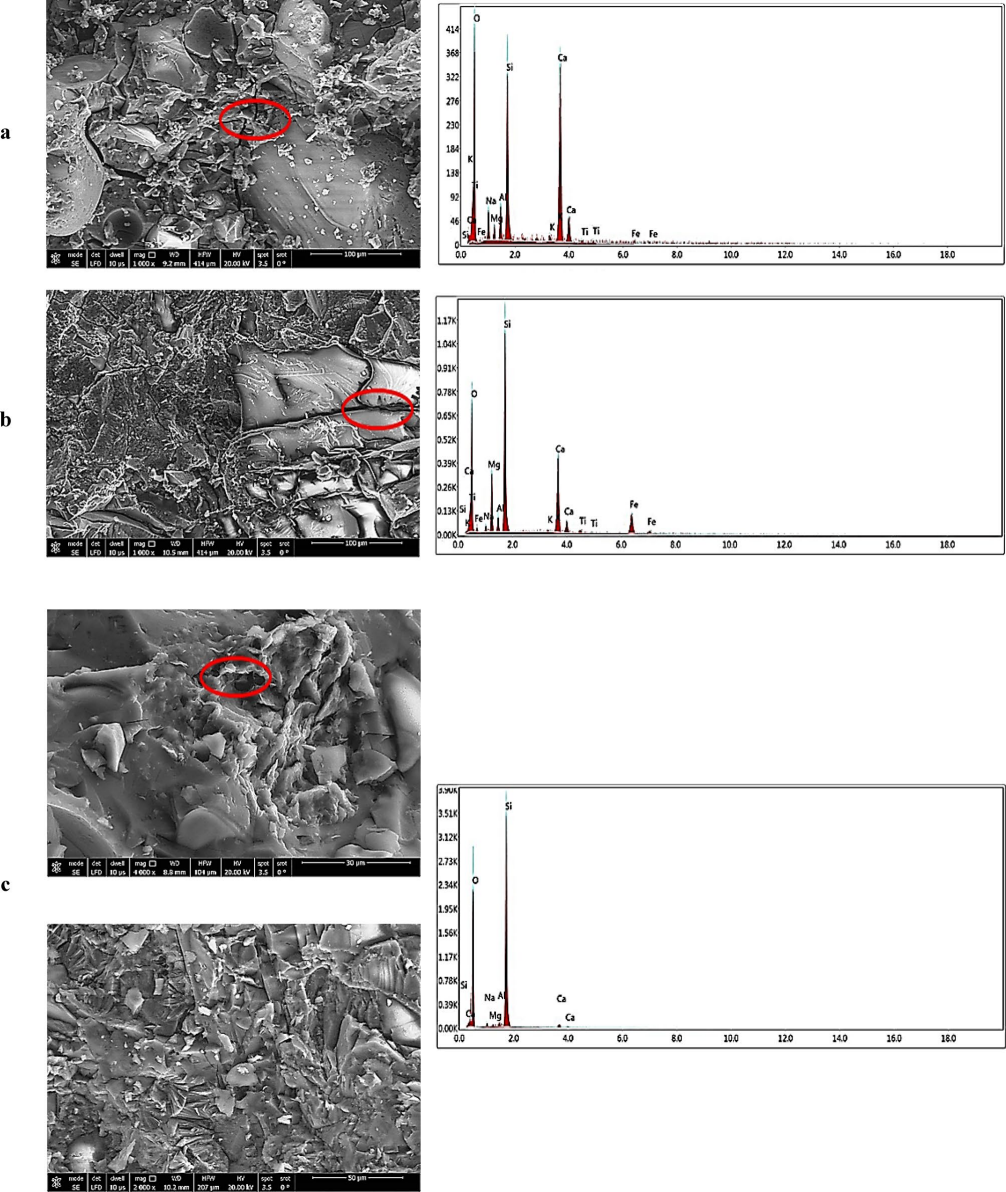

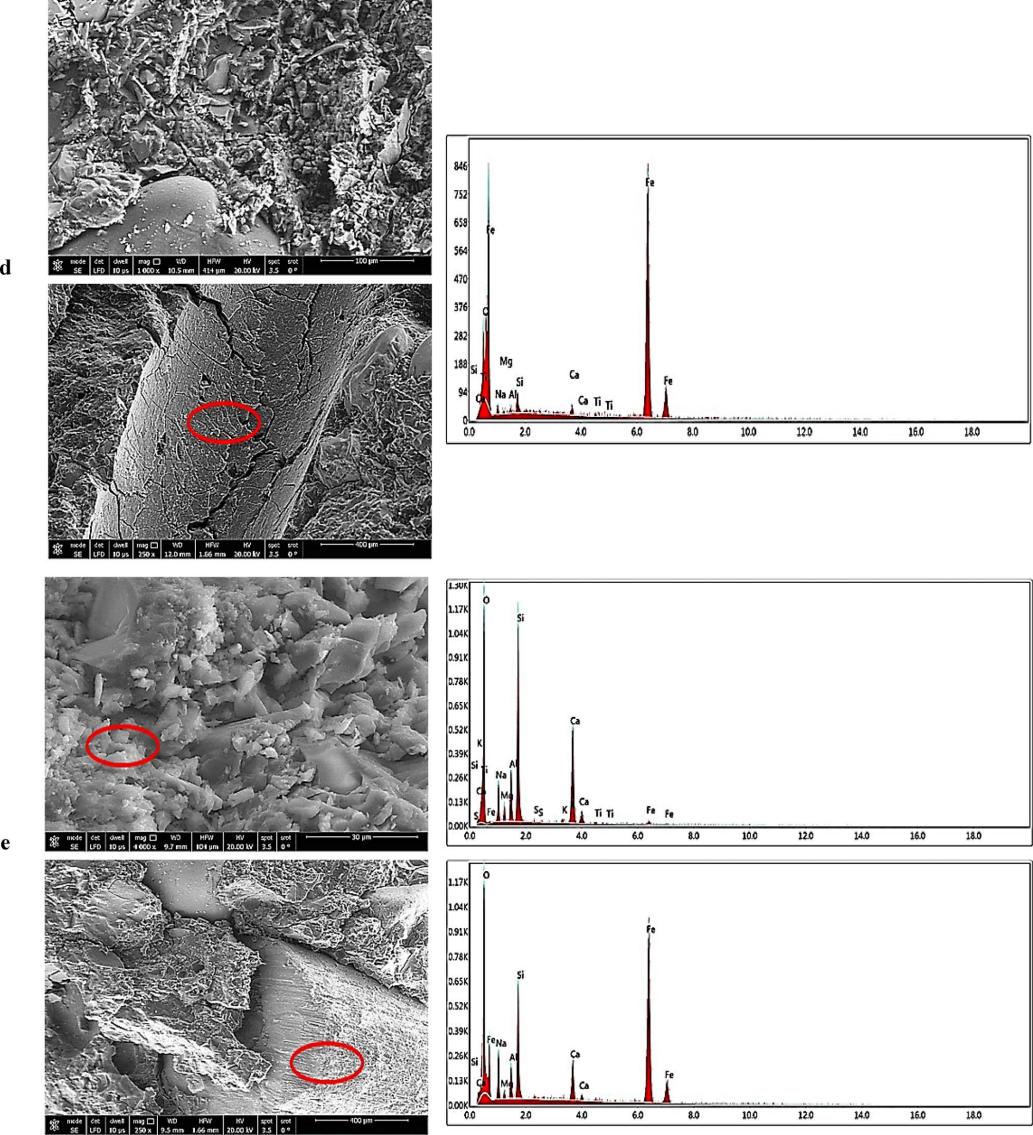
SEM images and EDX analysis of AASC samples after 28 days of curing: (**a**) MD, (**b**) MB, (**c**) MB-15G, (**d**) MB-15G-1 S, and (**e**) MB-10DK-1 S.

The SEM micrographs reveal progressive microstructural refinement across the AASC samples. Micrograph analysis of Sample MD (Fig. 9a) reveals a highly heterogeneous matrix with poorly bonded interfaces between dolomite aggregates and the AAS matrix. The ITZ, while distinct, appears underdeveloped and is characterized by the presence of microcracks and localized weak points. Dolomite aggregates interact minimally with the matrix, indicating low chemical compatibility. The matrix is characterized by significant porosity, with pore sizes ranging from 5 to 30 μm, and partially reacted GGBFS particles, contributing to reduced mechanical strength. EDX analysis reveals that Ca, Si, and O are the predominant elements, with high Ca zones around the dolomite and low Al content, indicating limited aluminosilicate gel formation and insufficient geopolymerization, consistent with the weak mechanical performance.

Sample MB (Fig. 9b), containing silicate-rich basalt aggregates, demonstrated improved stress distribution and chemical interaction. Its microstructure is more homogeneous with denser particle packing and predominant pores of ~ 5.2 μm. EDX reveals higher Si, Mg, and Fe with moderate Ca. The higher Si/Ca ratio suggests increased silicate-based gel formation, correlating with moderate enhancement in mechanical properties compared to MD.

In MB-15G (Fig. 9c), the matrix displays a dense and homogeneous geopolymeric gel (AAS gel, C-S-H). Bright angular particles indicate unreacted or partially reacted components. The ITZ is thin and well-bonded, while pores (~ 5–20 μm) are discrete, reflecting improved matrix packing. The inclusion of WGP promotes additional binding phases, enhancing gel formation and microstructural density. EDX shows exceptionally high Si with reduced Ca, confirming the presence of glass-derived silicate phases. These changes explain the substantial mechanical improvement over MD and MB.

MB-15G-1 S (Fig. 9d) highlights effective fiber-matrix interaction. The SF is surrounded by a dense, homogeneous matrix with reduced porosity and smaller isolated pores. Microcracks away from the fibers do not propagate through the ITZ, demonstrating crack-arresting capability. The synergistic effect of WGP and SF enhances gel formation and toughness. EDX shows high Fe in fibers and significant Si, Al, and Na in the surrounding matrix, confirming strong fiber integration and improved bonding.

MB-10DK-1 S (Fig. 9e) exhibits a highly homogeneous, dense matrix with two distinct gel phases: AASC gel (C-S-H) and N-(C)-A-S-H, forming a well-integrated aluminosilicate network. The ITZ with basalt aggregates is exceptionally developed, with minimal gaps and gradual transition, indicating extensive chemical bonding. Porosity is further reduced, and the continuous binding phase enhances cohesion and mechanical strength. The hybrid gel structure, formed through DK and GGBFS interaction, combines dense calcium-rich C-A-S-H gel with polymerized N-A-S-H gel, optimized by a Si/Al ratio of ~ 2.57. The observed Si/Al ratio facilitates the dissolution of aluminate and silicate species under alkaline conditions and their subsequent recombination into sialate (–Si–O–Al–O–) and polysialate (–Si–O–Al–O–Si–O–) linkages, which are stabilized by charge-balancing alkali cations^69^. This process promotes the simultaneous formation of calcium-rich gels and cross-linked aluminosilicate networks, leading to enhanced matrix densification and stronger interfacial bonding. These findings are in good agreement with previous studies^70,71^, which report that optimized Si/Al ratios in alkali-activated systems yield highly durable and chemically resistant binders. EDX confirms high Si, Al, and Na, supporting strong geopolymeric bonds. Minor Fe, S, and Ti reflect the complex DK composition. The combination of DK, SF, and basalt aggregates synergistically refines the microstructure, maximizes matrix density, and improves mechanical performance, representing the culmination of microstructural optimization across the sample series.

## Numerical simulation

To complement and validate the experimental findings, a numerical program was developed to simulate the mechanical behavior of AASC. The developed model is compatible with GPC and can be used for further studies to explore and predict its performance.

Experimental testing of concrete samples remains the most widely accepted method for evaluating concrete behavior. Standard tests on cubes, cylinders, and prisms are used to determine mechanical properties; however, variability in material properties, human error, and testing conditions can lead to variations in test results. To corroborate the experimental findings, a finite element modeling (FEM) program was implemented. Cubes, cylinders, and prisms were modeled in ABAQUS, incorporating experimentally obtained material properties, including compressive strength from cubes, tensile strength and elastic modulus from cylinders, and flexural strength from prisms.

**Fig. 10:**
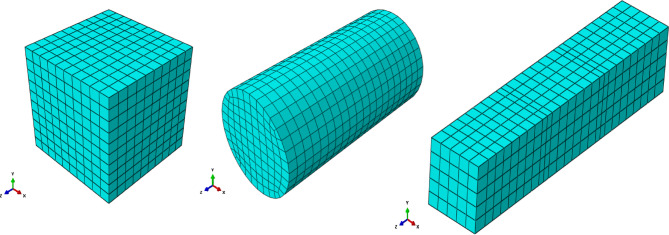
Finite element mesh of cube, cylinder, and prism specimens.

The models employed appropriate constitutive laws for GPC, such as the nonlinear CDP model, with boundary conditions and loading designed to replicate laboratory tests, in order to reproduce failure patterns and validate the simulations. The CDP model captures stiffness degradation, strength evolution, and post-peak response, enabling accurate finite element simulation at both material and structural levels. The numerical samples matched the experimental dimensions listed in Table 4: cubes (100 mm x 100 mm x 100 mm), cylinders (100 mm diameter * 200 mm height), and prisms (100 mm × 100 mm × 500 mm) as shown in Fig. 10.

### Material model

#### Concrete modeling

Improving the mechanical performance of reinforced concrete elements like beams, columns, and slabs continues to be a primary objective in contemporary structural engineering research^72^.

The nonlinear behavior of GPC was modeled using the Concrete Damaged Plasticity (CDP) model in ABAQUS. The CDP model was selected for its ability to simulate compressive crushing, tensile cracking, and stiffness degradation under monotonic loading. The material parameters required by the CDP model were obtained from the experimental stress–strain data generated in this study. The compressive and tensile branches of the constitutive law were calibrated by fitting the numerical response to the measured curves, ensuring agreement in elastic stiffness, peak strength, and post-peak softening behavior. When direct experimental information was not available, commonly adopted values from the literature were used as guidance and adjusted during calibration. The tensile and compressive equivalent plastic stresses, εt_pl_ and εc_pl_, represent the hardening factors that contribute to failure^73^. Table 6 shows the parameters for the plastic behavior that describe the CDP model.

**Table 6:** The used CDP model parameters in the FE analysis.

Dilation angle	35°
Eccentricity	0.1
fb0/fc0	1.16
K	0.667
Viscosity parameter	0

The dilation angle fundamentally signifies the relationship between volumetric strain and shear strain. The dilation angle can vary from 30° to 40°: A smaller dilation angle results in more brittle behavior, whereas a larger angle leads to increased ductility^74^. The second parameter of the CDP to be specified is the eccentricity of the plastic potential surface^75^. Additionally, the ratio of the biaxial to the uniaxial compressive yield stresses, fb0/fc0, and the ratio of the second stress invariant on the tensile meridian to that on the compressive meridian, K, must be supplied. The viscosity parameter must be established, as it is utilized in the viscoplastic regularization equations essential for enhancing the model’s convergence performance. The parameters were validated for compressive strengths in the range of 30–60 MPa and corresponding peak strains of approximately 0-0.0025, which encompass the material properties of the mixes used in this study.

Besides the previous parameters, the material behavior is defined by presenting the uniaxial tensile and compressive stress-strain curves in a tabular format. The damage mechanisms are characterized by the values of the damage parameters in tension and compression, dt and dc, which are supplied to the software in tabular format, each depending on the corresponding equivalent plastic strain. Damage variables for tensile (dt) and compressive (dc) often derived from experimental data or established codes (e.g., CEB-FIP Model Code 1990, fib Model Code 2010)^76–78^. The damage parameter dc can be defined as follows:

1 $$\:dc=\frac{(1-\eta\:c){\epsilon\:}_{c}^{in}{E}_{0}}{\sigma\:c+(1-\eta\:c){\epsilon\:}_{c}^{in}{E}_{0}}$$

where η_c_ represents the ratio of the plastic strain ε_pl_ to the inelastic strain ε_in_, and it can be assumed to be 0.7 based on earlier laboratory experiments. The correlation between elastic and plastic strains in the hardening regime can also be expressed by:

2 $$\:{\epsilon\:}^{in}=\:{\epsilon\:}_{c}-\frac{{\sigma\:}_{c}}{{E}_{0}}$$

3 $$\:{\epsilon\:}^{pl}={\epsilon\:}^{in}-\frac{{d}_{c}}{(1-{d}_{c})}\frac{{\sigma\:}_{c}}{{E}_{0}}$$

Similarly, the damage parameter related to tensile loading, dt, is determined by Eq. (4).

4 $$\:{d}_{t}=\frac{{\eta\:}_{t}{\epsilon\:}^{in}{E}_{0}}{{\sigma\:}_{c}+{\eta\:}_{t}{\epsilon\:}^{in}{E}_{0}}$$

**Fig. 11:**
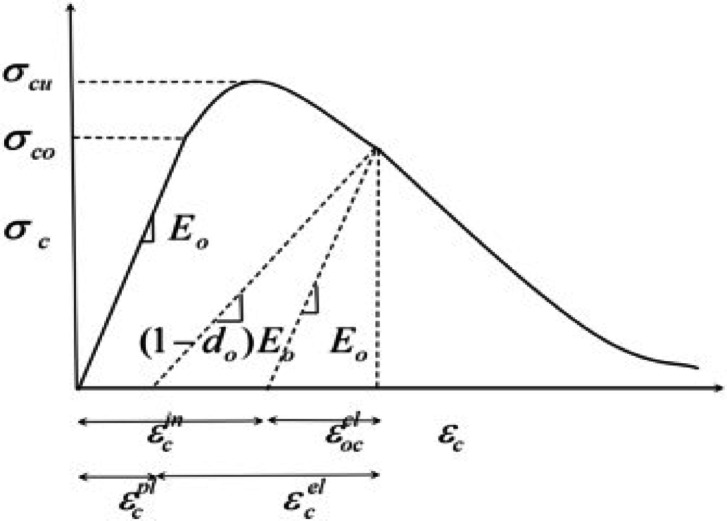
The concrete damage plasticity model^79^.

The values of d_t_ and d_c_ were calculated from the experimental stress–strain curves using the pervious equations, such that the damage evolution implemented in the model reproduces the measured mechanical response of the mixtures. Figure 11 shows the CDP model used in this study. It is important to assign the inelastic strain ε^in^ to a further extent to fit the ABAQUS settings. The maximum elastic stress of concrete should be placed in the first row of the compression part of the CDP model. Consequently, all initial strains ε_c_ were converted to inelastic strains ε^in^ to represent the inelastic behavior.

The bond between reinforcement and GPC is critical for structural performance, and the common assumption of a perfect bond has notable limitations, especially in advanced materials like geopolymers. A perfect bond was assumed between the reinforcement and the GPC matrix to maintain computational simplicity. However, this assumption can be problematic for GPC due to its unique microstructure and bond characteristics. GPC exhibits different bond-slip behavior compared to ordinary Portland cement (OPC) concrete, with bond strength and slip affected by factors such as bar diameter, embedment length, and interface roughness^80–82^. GPC may show more brittle failure and different crack patterns, which are not captured by perfect bond models^83,84^.

#### Element type and mesh

In this study, the concrete domain was discretized using three-dimensional eight-node brick elements with reduced integration (C3D8R). A maximum mesh size of 10 mm was selected for these elements. A mesh sensitivity study was carried out to ensure that the numerical response was not unduly affected by element size. Several mesh densities were analyzed and the differences in predicted peak load and load–displacement response were found to be small. The final mesh size used in the simulations represents a compromise between accuracy and computational efficiency.

#### Boundary conditions and load arrangements

**Fig. 12:**
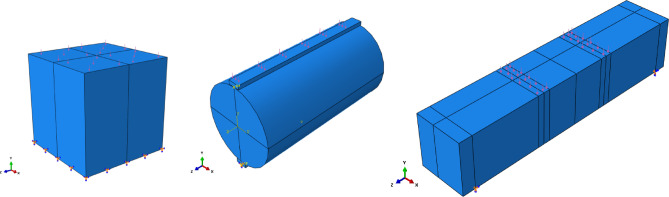
Boundary conditions and loading model for cube, cylinder, and prism specimens.

For compressive tests, cubic specimens (100 mm sides) were subjected to uniaxial loading, with fixed support at the base and displacement-controlled loading at the top surface. For tensile strength, a cylindrical specimen is loaded along its diameter until failure. Flexural tests employed prism specimens with simply supported conditions and two-point loading, as shown in Fig. 12.

### Results of the finite element simulation model

The calibrated CDP model was validated by comparing the predicted and measured responses of the GPC specimens. Good agreement was obtained in terms of ultimate loads and the characteristic failure modes for all specimen types. This consistency confirms the reliability of the developed finite element modeling approach and its capability to accurately simulate the mechanical behavior of AASC. The following section presents and discusses the finite element results for each model, highlighting their correlation with the corresponding experimental outcomes.

#### FEM compressive strength predictions

**Fig. 13:**
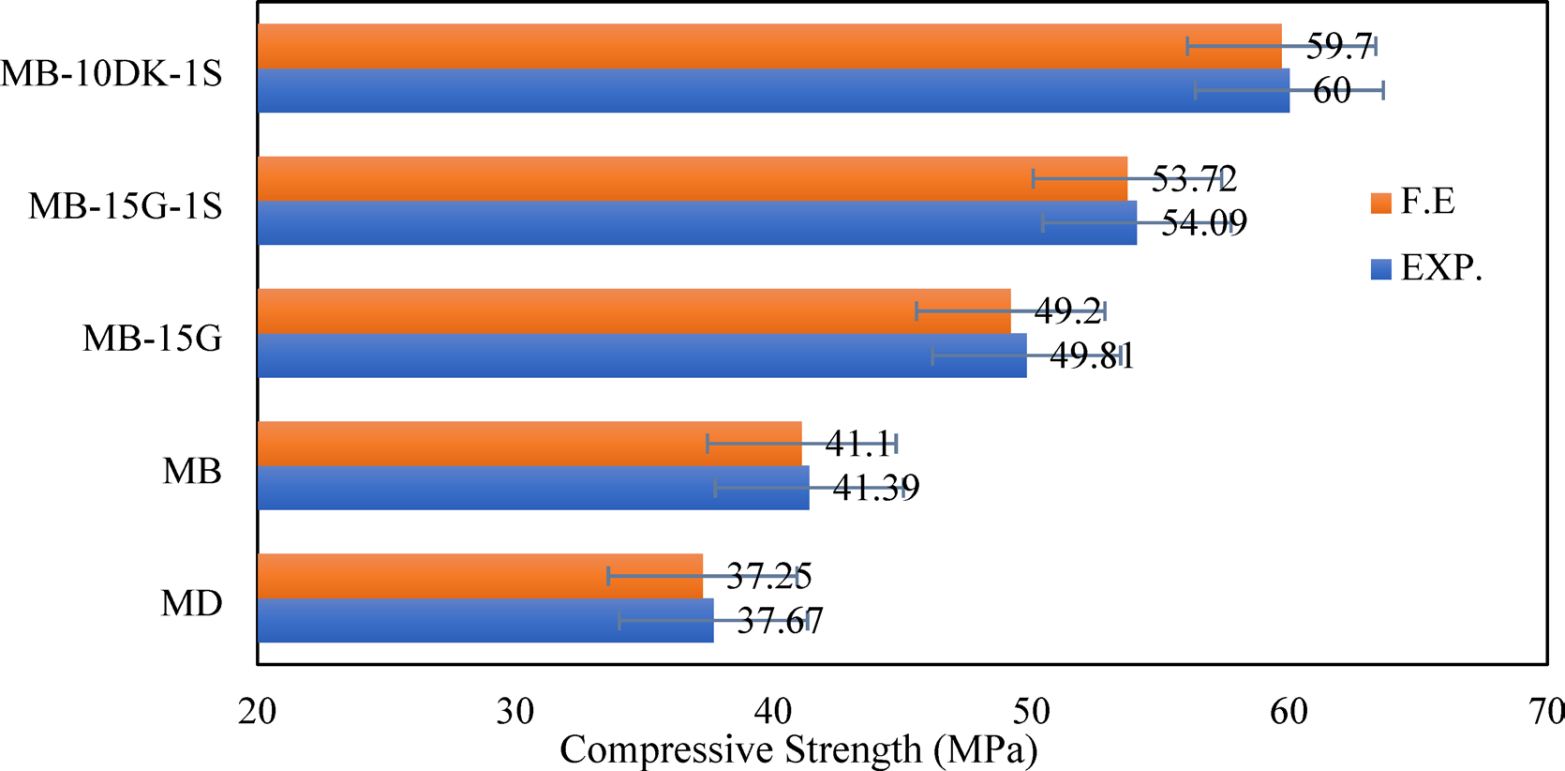
Comparison between experimental and finite element results in terms of compressive strength from cube tests.

Table 7 shows the comparison between compressive strengths obtained from FEM predictions and the corresponding experimental results for the cube specimens. It also presents the ratio of FEM to experimental ultimate load values. The results indicate that the average ratio between FE and experimental compressive strength is 99.2%, with a standard deviation of 0.33%, demonstrating the high reliability of the FEM results. Additionally, the comparison between experimental and FEM results in terms of failure loads for cube specimens is illustrated in Fig. 13. Figure 16 presents the numerical and experimental cracking patterns, showing that the finite element cracking pattern closely matches the experimental observations.

**Table 7:** Comparison between compressive strength from cube tests for Exp. and F.E.

Specimens	Compressive strength (MPa)		% (F.E./EXP.)
	EXP.	F.E.	
MD	37.67	37.25	98.9
MB	41.39	41.1	99.3
MB-15G	49.81	49.2	98.8
MB-15G-1S	54.09	53.72	99.3
MB-10DK-1S	60	59.7	99.5
		Average	99.2
		Standard deviation	0.33

#### FEM splitting tensile strength predictions

**Fig. 14:**
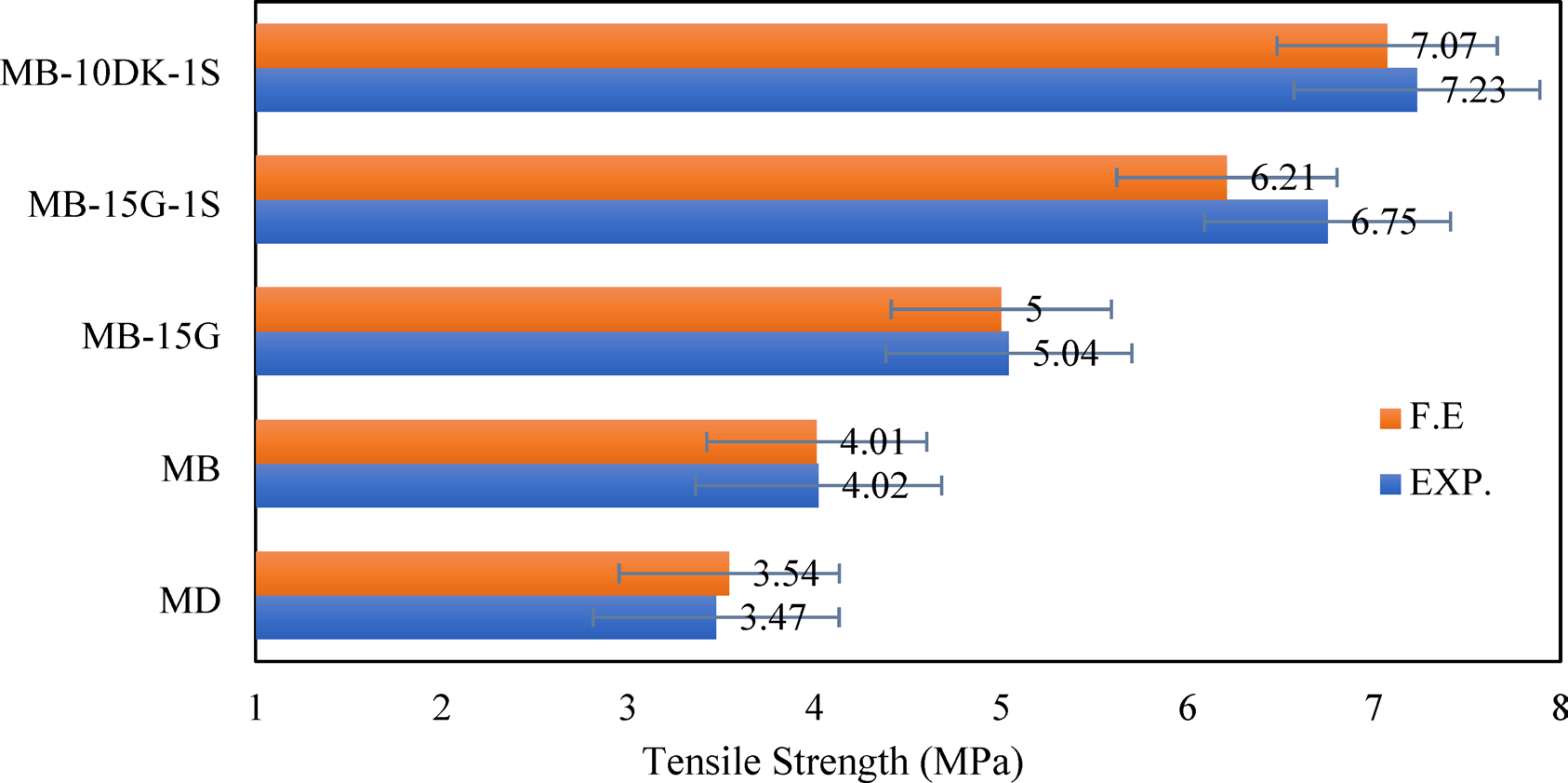
Experimental versus finite element in terms of tensile strength from cylinder tests.

The experimental and finite element results for tensile strength of cylinder specimens are compared in Fig. 14. The results indicate that the finite element model aligns well with the experimental outcomes, confirming its reliability in predicting splitting tensile strength up to failure. Table 8 presents the splitting tensile strength values at failure for both FEM predictions and experimental tests. The average ratio between FEM and experimental results is 98.15%, with a standard deviation of 3.76% across all specimens. Figure 14 illustrates the comparison of splitting tensile strength values, while Fig. 16 shows the numerical and experimental fracture patterns, demonstrating that the finite element cracking patterns closely correspond with the experimental observations.

**Table 8:** Comparison between tensile strength from cylinder tests for Exp. and F.E.

Specimens	Splitting tensile strength (MPa)		% (F.E./EXP.)
	EXP.	F.E.	
MD	3.47	3.54	102.02
MB	4.02	4.01	99.75
MB-15G	5.04	5	99.2
MB-15G-1S	6.75	6.21	92
MB-10DK-1S	7.23	7.07	97.78
		Average	98.15
		Standard deviation	3.76

#### FEM flexural strength test simulations 

**Fig. 15:**
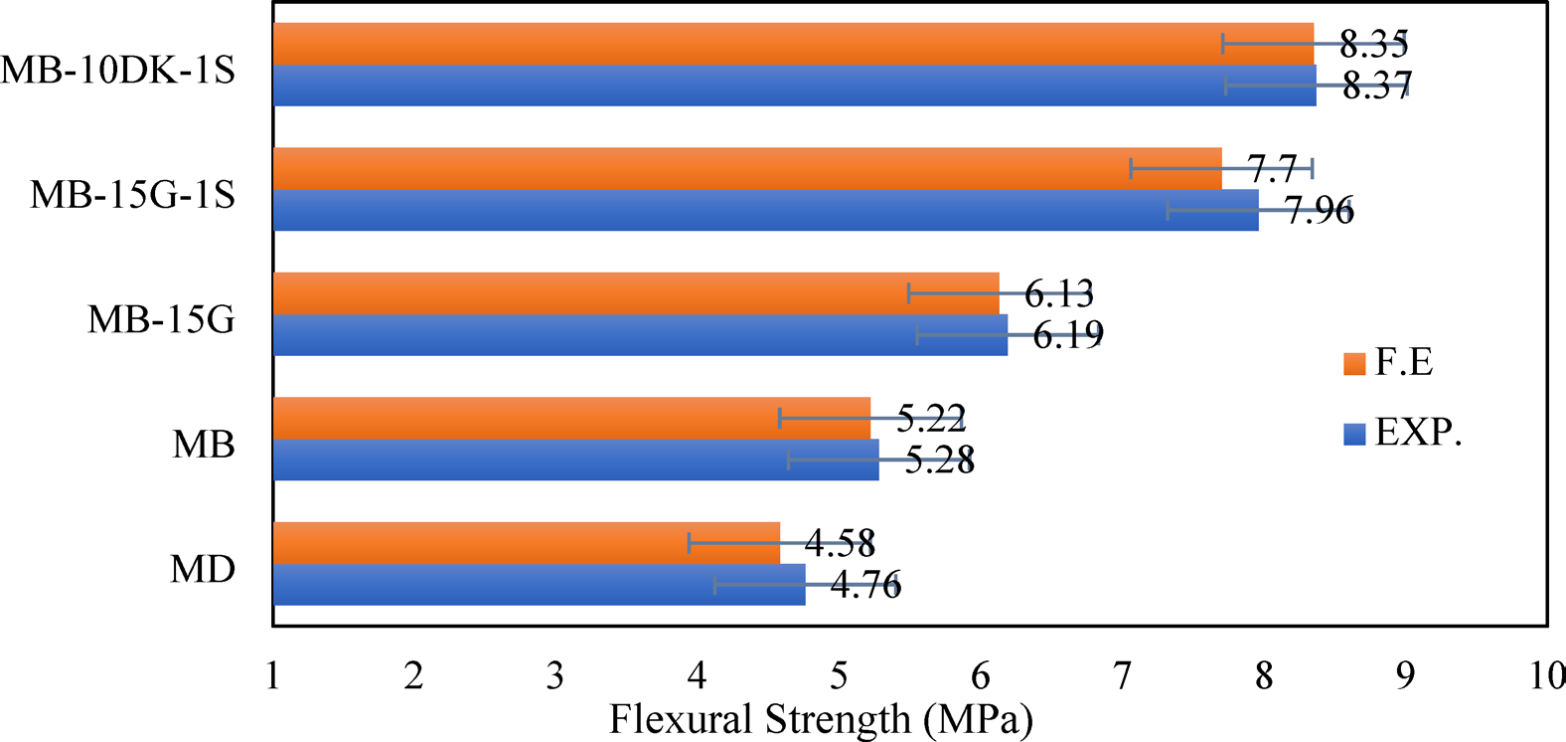
Experimental versus finite element in terms of flexural strength from prism tests.

**Fig. 16:**
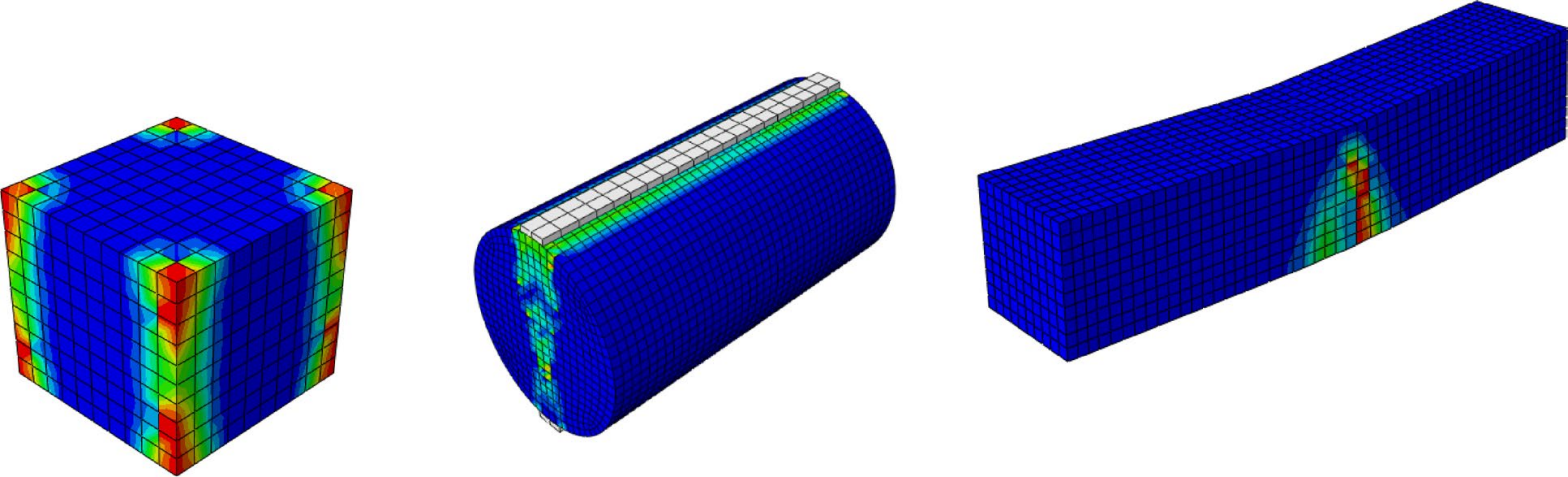
The numerical cracking patterns of all tested specimens.

The study demonstrates that FEM analysis provides reasonably accurate predictions of flexural strength compared to experimental prism tests, with deviations within an acceptable range (< 2%), as summarized in Table 9. The FEM predictions were slightly lower (1–4%) than the experimental values, likely due to idealizations in material modeling. Figure 15 presents the FEM and experimental results at the ultimate load, while Fig. 16 illustrates the corresponding numerical and experimental cracking patterns, showing close agreement between them.

**Table 9:** Comparison between flexural strength from prism tests for Exp. and F.E.

Specimens	Flexural strength (MPa)		% (F.E./EXP.)
	EXP.	F.E.	
MD	4.76	4.58	96.22
MB	5.28	5.22	98.86
MB-15G	6.19	6.13	99.03
MB-15G-1S	7.96	7.7	96.73
MB-10DK-1S	8.37	8.35	99.76
		Average	98.12
		Standard deviation	1.55

This numerical study successfully demonstrated the potential of finite element modeling to determine the mechanical properties of GPC. The results align with experimental findings, highlighting the feasibility of using numerical tools for preliminary design and analysis of AASC structures. This model will be utilized to extend the study on the flexural behavior of GPC.

## Parametric study

Following the successful validation of the nonlinear finite element model against experimental results, a comprehensive parametric study was conducted to extend the investigation from material-level behavior to structural-scale performance. The primary objective of this study is to evaluate the influence of different AASC mix compositions on the flexural response of reinforced concrete beams, while simultaneously assessing the robustness and predictive capability of the validated Concrete Damaged Plasticity (CDP) model. Such an approach enables systematic assessment of key performance indicators, including load-carrying capacity, stiffness, crack initiation and propagation, and overall ductility, which are difficult to fully capture through experimental testing alone.

**Fig. 17:**
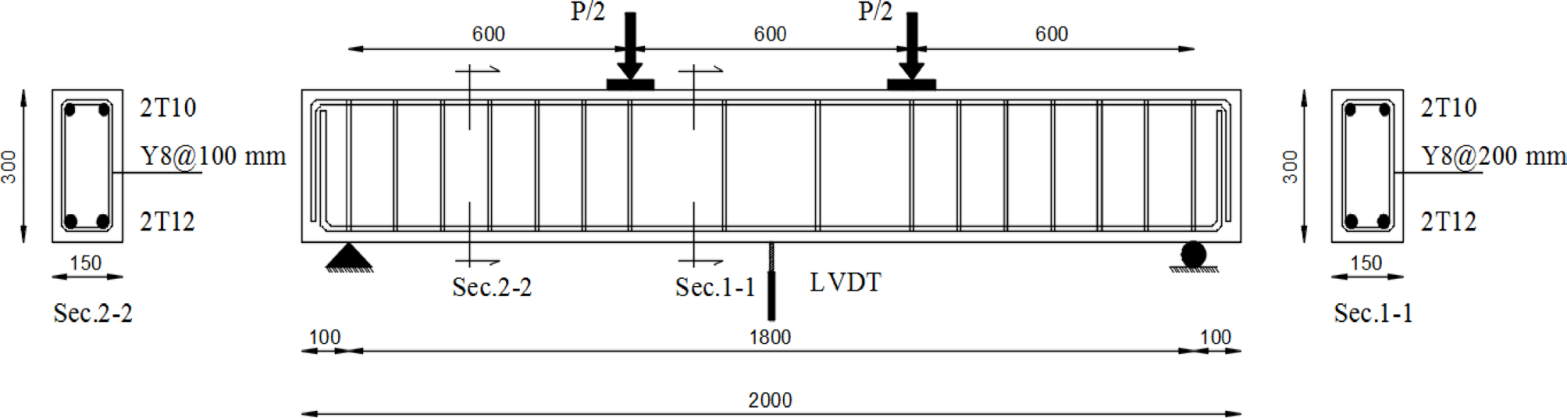
Details of the tested beams.

Following this validation, a comprehensive parametric study was carried out to investigate the flexural behavior of reinforced AASC beams. Five beam specimens were numerically analyzed to evaluate the structural response of AASC, as summarized in Table 10. The beam models were constructed using geometrical dimensions and reinforcement details derived from the experimental program, as illustrated in Fig. 17. The main variable in the parametric study was the concrete mix composition of each specimen, as listed in Table 10, while the remaining parameters were kept constant to isolate the effect of mix design. The material properties were determined on the basis of experimentally obtained data.

The total beam length was 2000 mm with a clear span of 1800 mm, a width of 150 mm, and an overall depth of 300 mm. A four-point static bending configuration was simulated, with two concentrated loads applied to generate a constant moment region at midspan.

**Table 10:** Details of the tested specimens.

Specimen ID	Mix	Top reinforcement	Bottom reinforcement	Stirrups
B1	MD	2 Ф 10	2 Ф 12	Ø 8 @ 200 mm
B2	MB			
B3	MB-15G			
B4	MB-15G-1S			
B5	MB-10DK-1S			

### Modelling of the beams in ABAQUS software

The beams modeling in ABAQUS include the simulation of GPC, longitudinal reinforcements, shear stirrups, boundary conditions, and applied load. All beam models were developed using solid elements for concrete and embedded truss elements for reinforcement.

All reinforcement bars and stirrups are assigned a linear elastic material behavior up to failure, with no damage criteria applied. The properties of the steel reinforcement bars are provided in Table 11. The elastic characteristics are assumed to include a Young’s modulus of 200 GPa and a Poisson’s ratio of 0.3.

**Table 11:** Mechanical properties of the used steel reinforcement bars .

Elastic modulus (MPa)	Yield stress (MPa)	Ultimate stress (MPa)	Poisson’s ratio	Density (kg/m^3^)
200,000	400	600	0.3	7850

**Fig. 18:**
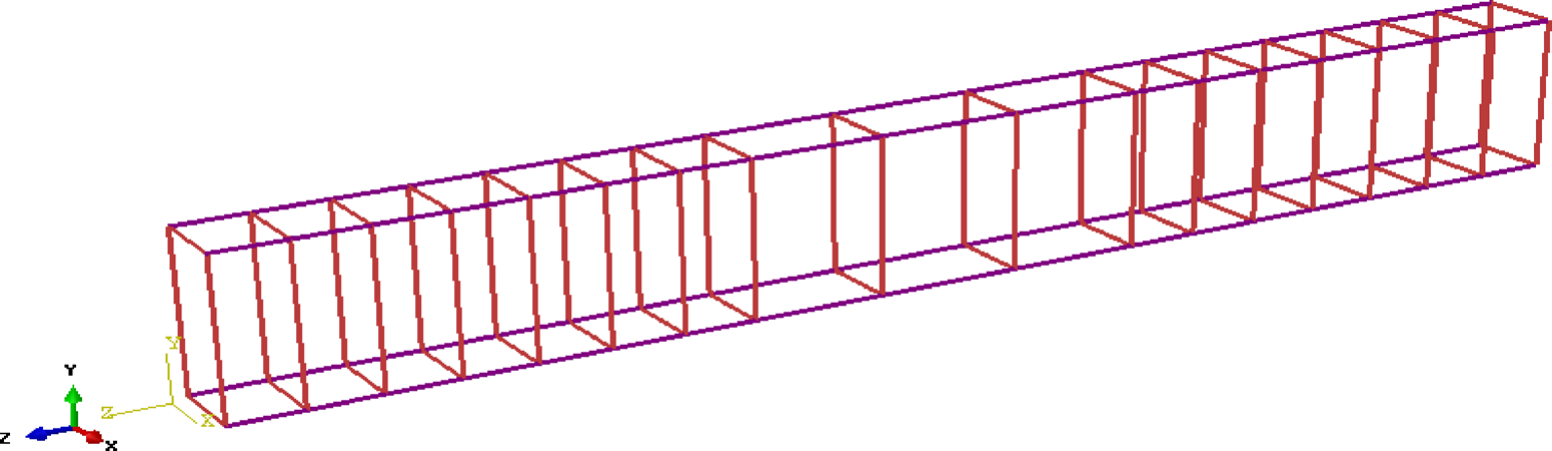
Details of reinforcement of the tested specimens.

**Fig. 19:**
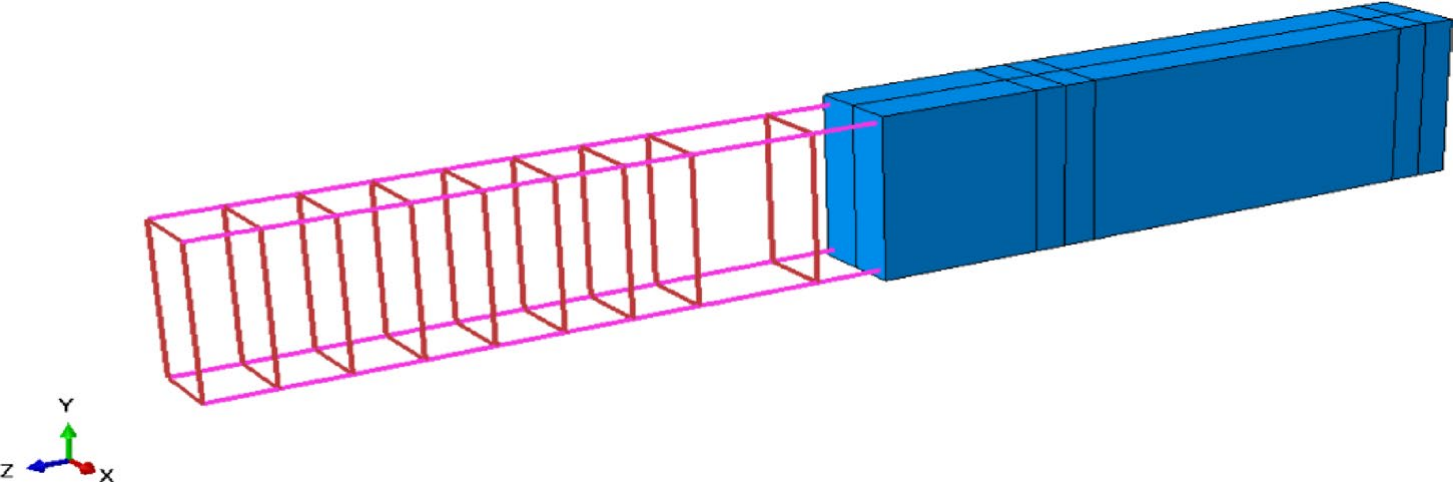
Host and embedded regions in the beam model in the FE software.

The embedded-region constraint used in the beam models assumes a perfect bond between the reinforcement and the concrete, and thus bond–slip effects are not explicitly modeled. Future work will incorporate bond–slip relationships to capture potential differences in interfacial behavior between AASC/geopolymer and OPC concretes. This method involves the use of embedded elements, which are a set of elements placed within host elements. It is primarily used to simulate the bond between the surrounding concrete and the reinforcement bars. A fully bonded condition is represented by constraining the translational degrees of freedom of the embedded elements (i.e., the reinforcement bars) to match those of the host elements (i.e., the concrete), as illustrated in Fig. 19.

The contact behavior was defined in terms of both tangential and normal interactions to account for friction and separation. The tangential behavior used a penalty friction formulation with a coefficient of 0.5, and the normal behavior employed a hard contact pressure-over-closure relationship.

**Fig. 20:**
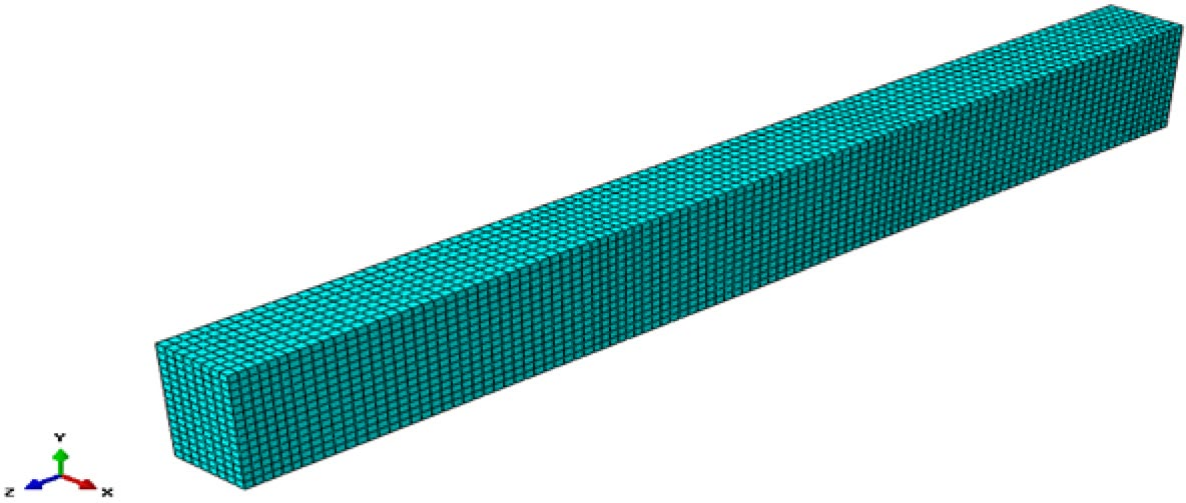
Meshing of the beams in the FE software.

The element meshing and constitutive material models all have an impact on how accurate the findings are. Therefore, these parts are precisely investigated. In this study, the concrete was represented by eight-node brick elements with reduced integration (C3D8R). A maximum mesh size of 20 mm was selected for these elements. The reduced integration approach avoided the need for higher-order solid elements, while still maintaining the reliability of the measured responses. This element type also addressed hourglass issues that frequently arise with continuum linear solid elements. A regular structured hexahedral mesh was employed. The reinforcement bars were modeled using three-dimensional truss elements (T3D2) in linear order, with a maximum mesh size of 20 mm, as shown in Fig. 20.

**Fig. 21:**
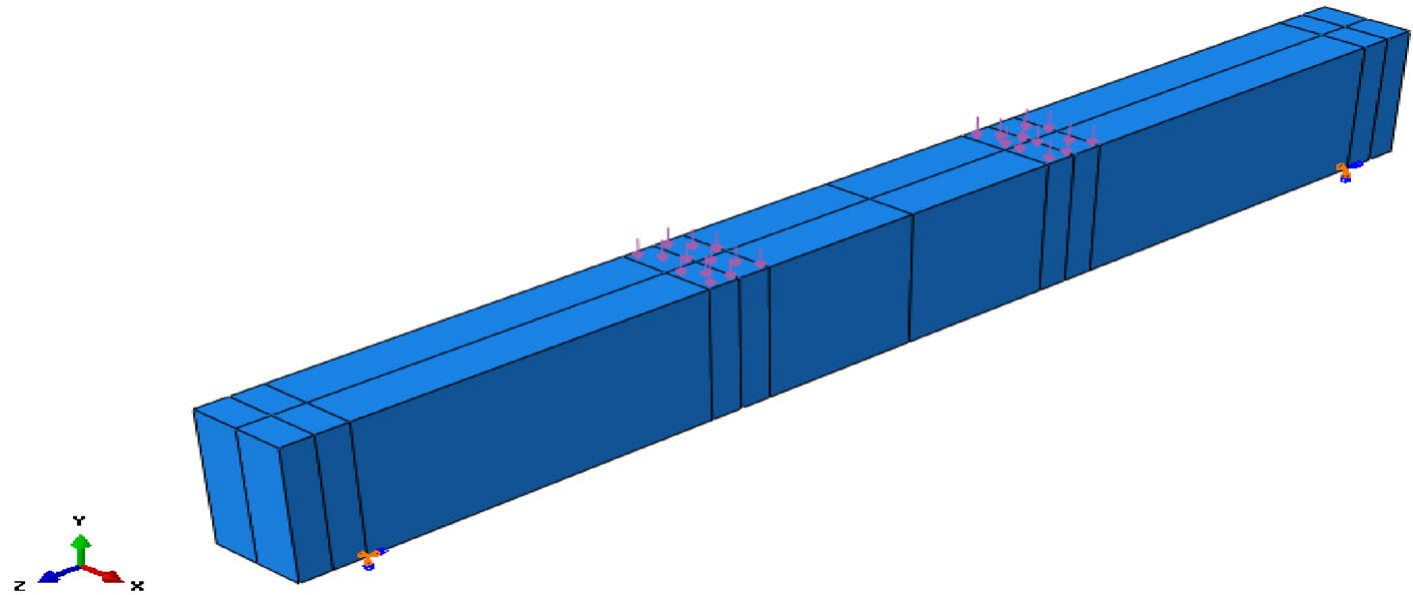
Boundary conditions and loading of the beam model in the FE software.

As illustrated in Fig. 21, the external loads are represented as a proportional pressure distributed over a 100 mm × 150 mm area on the concrete beam’s top surface at the specified load sites. Boundary condition approaches are used in the model to support the tested beams. The imposed boundary conditions for each specimen are shown in Fig. 21. Translations and rotations were completely restrained to simulate a hinged support (Ux = Uy = Uz = URy = URz = 0), whereas they were restricted appropriately to simulate a roller support (Ux = Uy = URy = URz = 0).

### Results of the parametric study

**Fig. 22:**
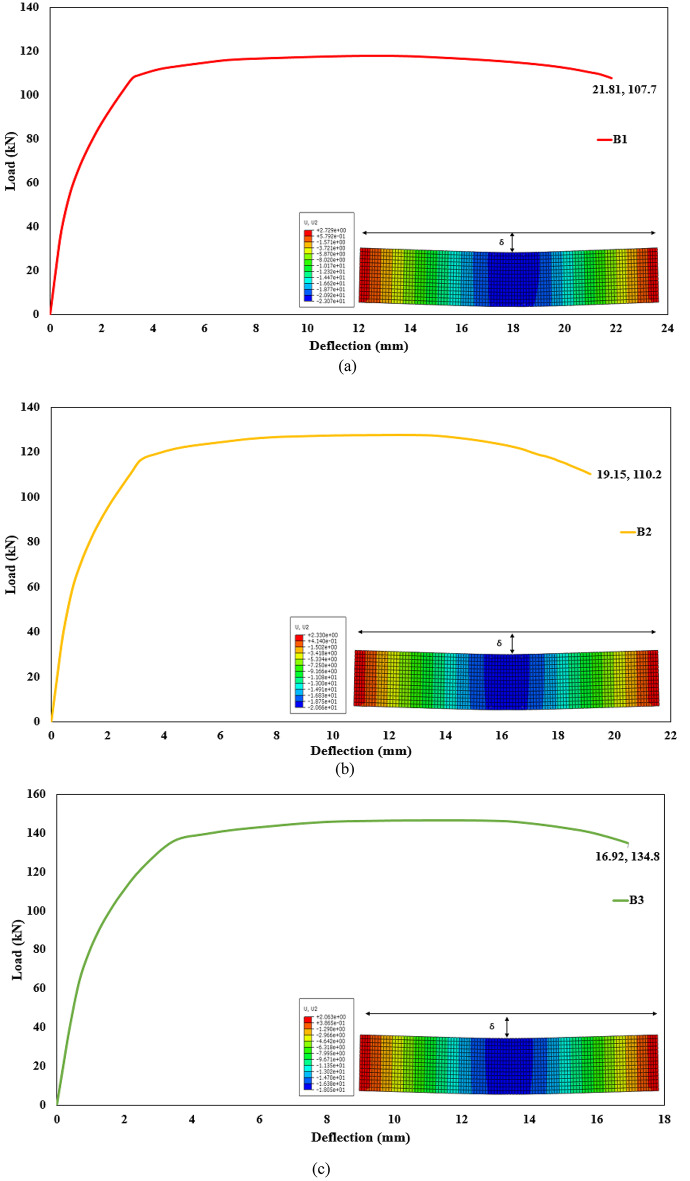

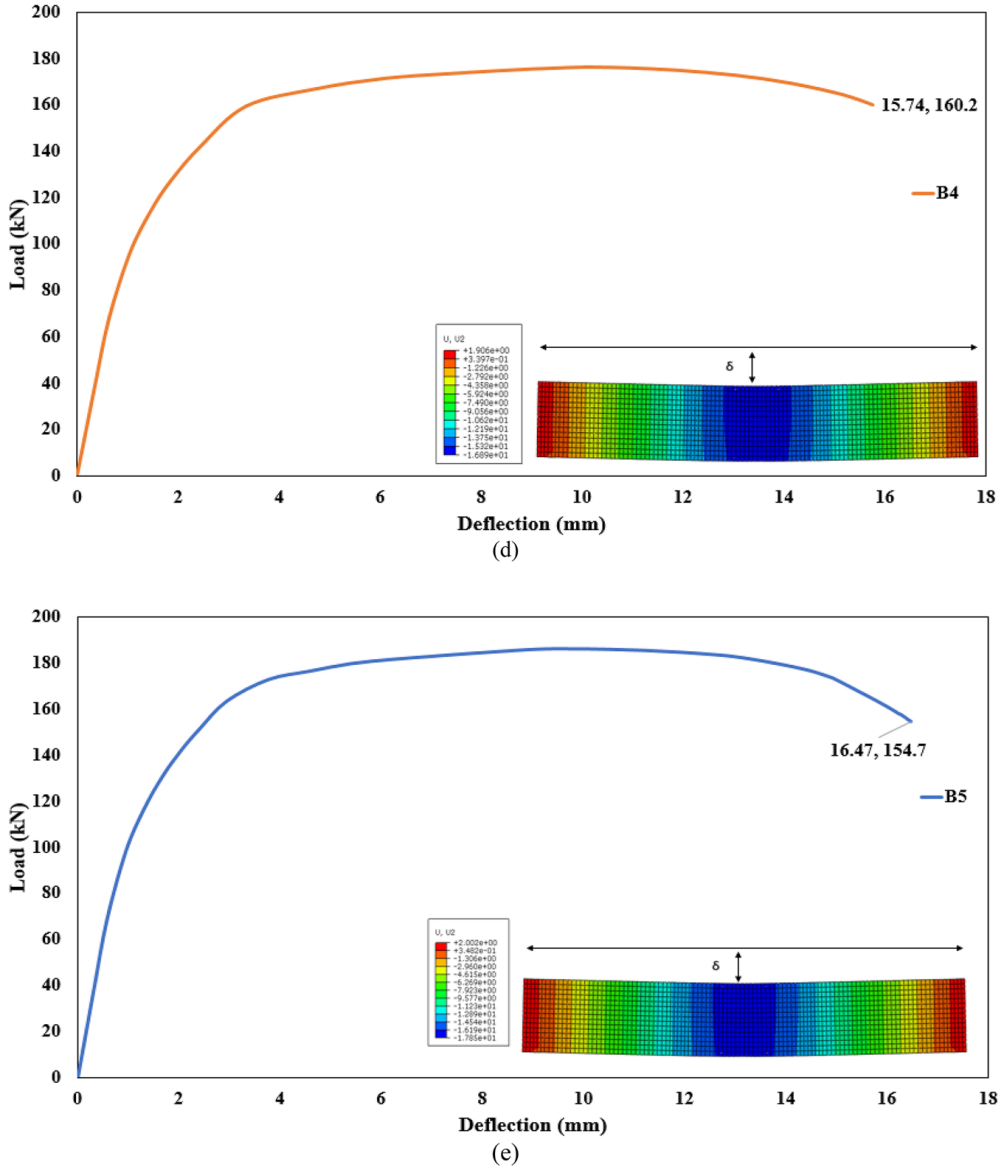
Load-deflection relationships for the tested beams obtained from finite element analysis: (**a**) B1, (**b**) B2, (**c**) B3, (**d**) B4, and (**e**) B5.

**Fig. 23:**
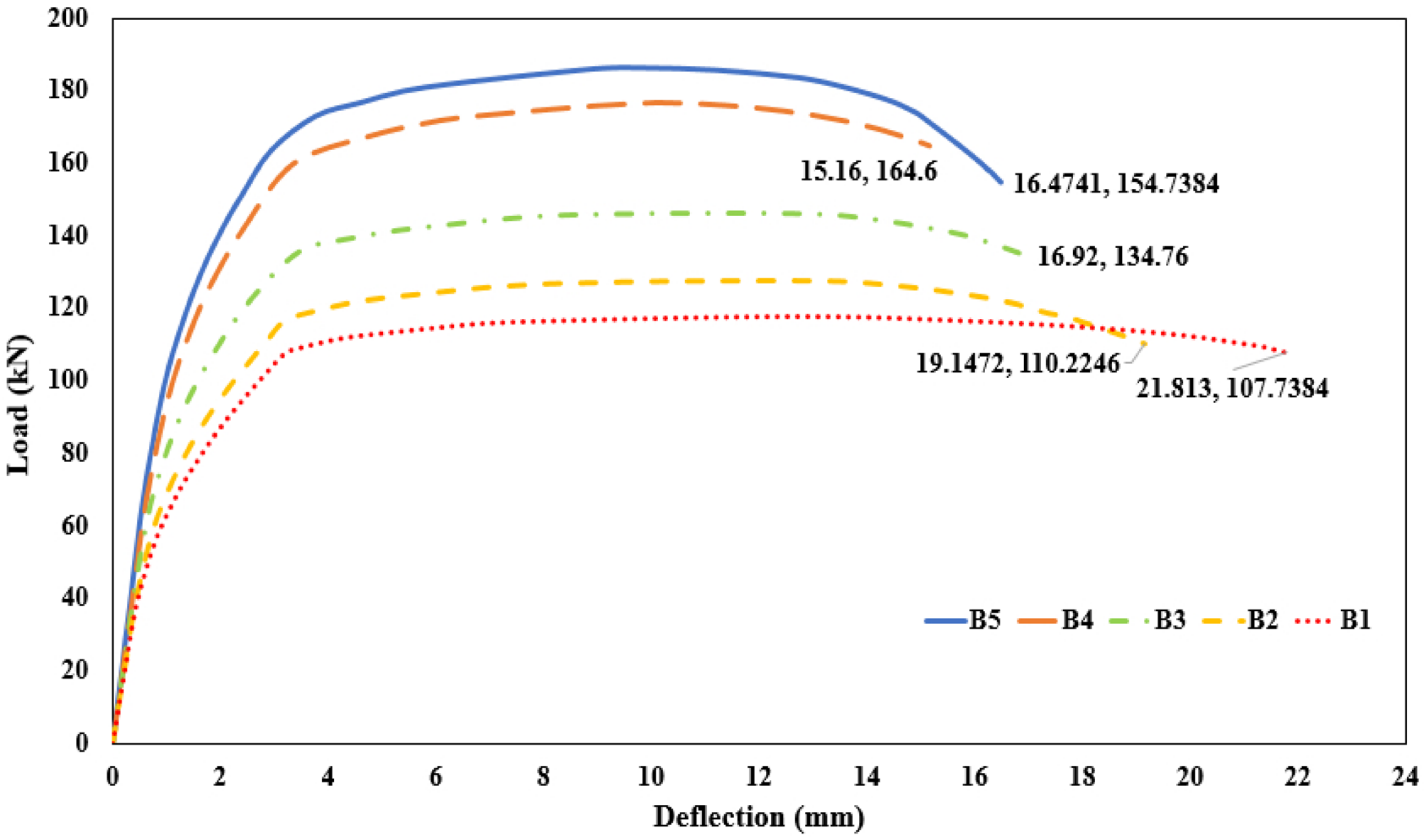
Comparison of load-deflection relationship for the tested beams.

The results of the current study will be discussed in this section for all beams models. The beam results reported in this study are obtained exclusively from numerical simulations. No full-scale beams were physically tested. The structural responses presented are therefore purely numerical and are based on the finite element model incorporating the previously validated material constitutive relationships. This section illustrates the effect of using different proportions of mixes on the flexural behavior of the AASC beams. A summary of study results, including the ultimate and failure load. Also, the mid-span deflection value, is presented in Table 12. It should be noted that the ultimate load of the beam is defined as the maximum load reached in the numerical load–deflection curve prior to any subsequent load reduction. The results are also presented in terms of the load-deflection for all beams, as shown in Fig. 22. Furthermore, Fig. 23 shows a comparison of the load-deflection curves for AASC beams with different proportions of mixes.

#### Effect of the basalt on the concrete mix in AASC

Basalt-based beams (B2, B3, B4, B5) exhibited higher stiffness and peak load compared to the dolomite-based beam (B1). For instance, beam B2 exhibited an 8.23% higher ultimate load than beam B1. The ultimate load of beam B2 was 127.6 kN. Similarly, beam B3 demonstrated an 24.2% higher ultimate load than beam B1, with the ultimate load of 146.4 kN. Further, beams B4 and B5 respectively, showed ultimate load increases of 49.7%, and 58.02% compared to beam B1. The ultimate loads for these beams were 176.5 kN and 186.3 kN, respectively, as shown in Table 12.

**Table 12:** Values of ultimate, failure load and mid-span deflection for tested beams.

Beam ID	Ultimate load (kN)	Deflection at ultimate (mm.)	Failure load (kN)	Deflection at failure (mm.)
B1	117.9	12.76	107.7	21.8
B2	127.6	12.16	110.2	19.15
B3	146.4	11.46	134.7	16.92
B4	176.5	10.22	164.6	15.16
B5	186.3	9.77	154.73	16.47

#### Effect of the addition WGP on the concrete mix in AASC

The addition of WGP with basalt in AASC can enhance ductility and ultimate load capacity. Figure 22 illustrates the effect of adding WGP with a ratio of 15% on beams B3 and B4. Beam B3 with WGP addition increased the ultimate load capacity by about 24.2%, while beam B4 with WGP addition and SF increased it by about 49.7%, both compared to reference beam B1 without WGP.

#### Effect of the addition of SF on the concrete mix in AASC

The addition of SF to AASC beams significantly enhances flexural strength, ductility, and crack control. The ultimate load and ductility of steel fiber reinforced AASC beams are higher compared to those of ordinary AASC beams. Increasing SF content in beam B4 (1% SF) led to an increase in the ultimate load of the beam by 49.7% compared to B1 without SF. Additionally, beam B5 (1% SF + basalt + 10% DK) showed the highest ultimate load capacity (186.3 kN), as shown in Table 12.

#### Effect of the addition of DK on the concrete mix in AASC

The inclusion of DK with a ratio of 10% reduced deflection due to a reduction of microcracking and improved geopolymerization, as illustrated in Fig. 23. The vertical deflection at ultimate of beam B5 was reduced by 23.4% compared to beam B1 without DK. The vertical deflections at ultimate for these beams were 9.77 mm and 12.76 mm, respectively, as shown in Table 12.

### Failure modes of the tested beams

**Fig. 24:**
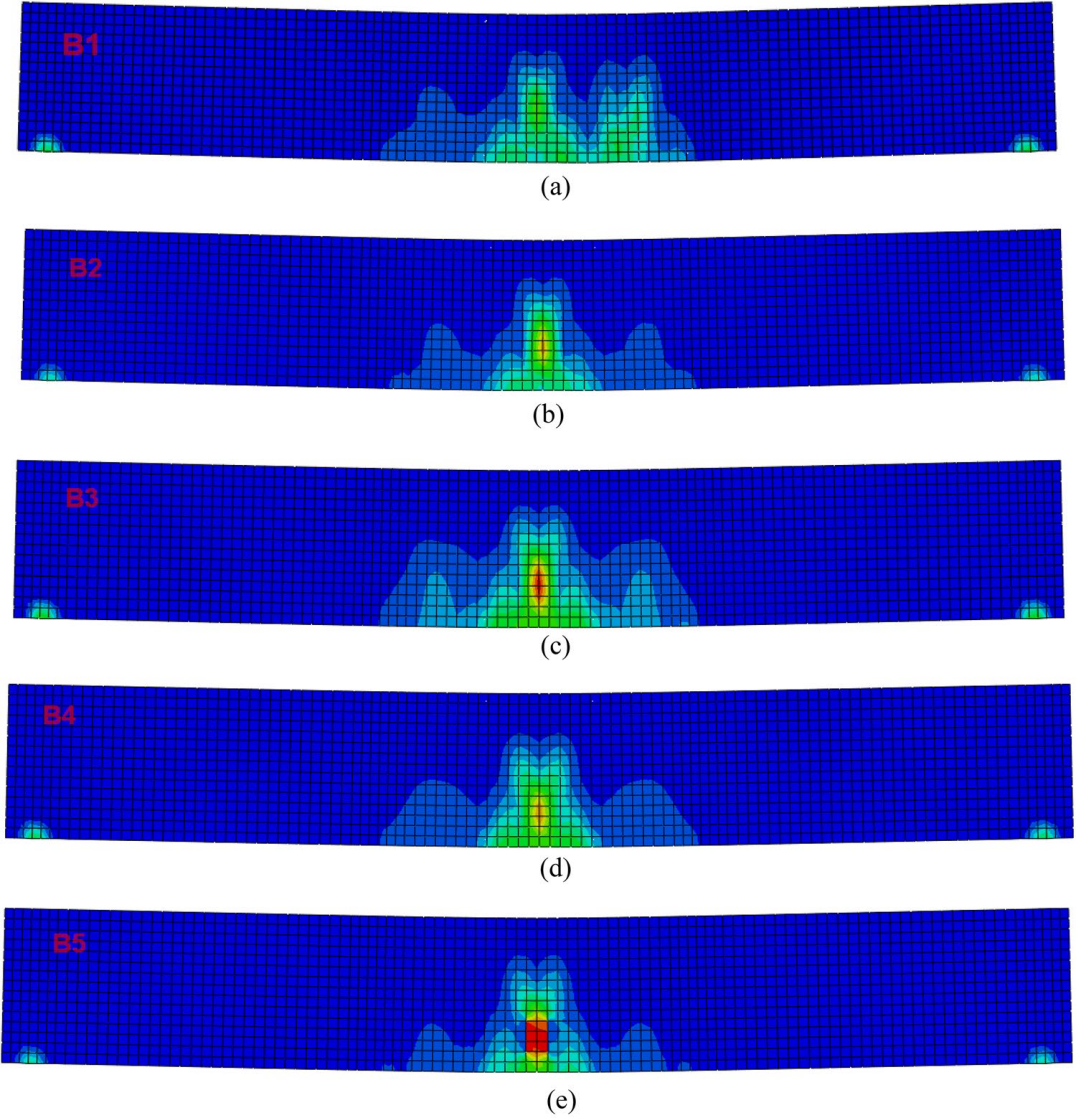
Failure mode for tested beams obtained from finite element analysis: (**a**) B1, (**b**) B2, (**c**) B3, (**d**) B4, and (**e**) B5.

Figure 24(a–e) shows the final failure modes of beams B1–B5. All beams primarily exhibited flexural failure, with cracking initiating in the tensile zone at mid-span and propagating upward as the applied load increased. In Beam B1 (MD: control AASC mix with crushed dolomite aggregate), the crack spacing was relatively large and failure was governed by a single dominant mid-span crack, reflecting the lower tensile resistance and stiffness of the control mix.

Beam B2 (MB: AASC with basalt aggregate) developed a greater number of flexural cracks with reduced spacing compared with B1, indicating the beneficial effect of basalt aggregate on stiffness and tensile crack distribution. Beam B3 exhibited a more distributed cracking pattern with an increased number of flexural cracks. This indicates enhanced ductility and a higher energy absorption capacity due to the incorporation of WGP, allowing the beam to undergo significant deformation before failure.

In Beams B4 (MB-15G-1 S) and B5 (MB-10DK-1 S), which included 1% steel fibers (SF) in addition to modified binder compositions, more widespread cracking was observed. Steel fibers contributed to crack bridging and redistribution, leading to finer crack networks before localization. Beam B5, containing 10% DK instead of WGP, showed concentrated cracking, consistent with its higher load-carrying capacity.

No dominant diagonal shear cracks were observed in any specimen, confirming that failure in all beams was governed by flexure rather than shear. Overall, the failure modes in Fig. 24 correlate well with the load–deflection responses and clearly demonstrate the influence of aggregate type, WGP and DK replacement, and steel fiber addition on crack formation and ultimate failure behavior. As a result, all types of concrete beam failure modes can be identified using the FEM.

## Conclusions

This study presents a comprehensive, multi-level evaluation of the mechanical behavior of Alkali-Activated Slag Concrete (AASC) incorporating both recycled and supplementary cementitious materials. AASC mixtures were developed with steel fibers (SF) combined with either recycled waste glass powder (WGP) or dealuminated metakaolin (DK), along with two types of coarse aggregates (dolomite and basalt), to enhance their structural response. Through an integrated program combining experimental testing, numerical modeling, and parametric simulations, the research investigates the mechanical properties of these mixtures, including compressive strength, splitting tensile strength, flexural capacity, modulus of elasticity, and stress–strain behavior. Complementary microstructural analyses using SEM–EDX provided deeper insight into matrix densification, the formation of reaction products, and the mechanisms responsible for the observed improvements in mechanical performance. To validate the experimental observations, finite element models of cubes, cylinders, and prisms were developed in ABAQUS using the measured material properties, demonstrating strong agreement between simulated and experimental results. The validated model was subsequently used to conduct a parametric study on AASC beams under four-point bending, enabling a broader assessment of flexural behavior under different mix compositions. The main findings of the study are summarized as follows:

- The choice of coarse aggregate plays a critical role in structural performance. Basalt aggregates exhibit superior chemical compatibility with the geopolymer matrix compared to dolomite, resulting in a more refined Interfacial Transition Zone (ITZ) and enhanced overall compressive strength.
- The incorporation of 10% DK significantly enhances the mechanical properties of AASC. This improvement is attributed to the high reactivity of DK, which promotes the formation of a denser N-(C)-A-S-H gel matrix, effectively filling micro-voids and refining the pore structure.
- While the addition of WGP offers environmental benefits, its combination with 1% steel fibers is essential to mitigate the inherent brittleness of the AASC matrix. The fibers provide effective crack-bridging mechanisms, transforming the failure mode from sudden brittle crushing to a more ductile, energy-absorbing structural response.
- SEM-EDX analysis confirmed that the observed superior mechanical trends are directly linked to the microstructural densification provided by the DK and SF. A direct correlation exists between the reduction in observable micro-cracks in the ITZ and the increased splitting tensile and flexural capacities.
- Optimized mixes achieve higher ultimate loads and lower deflections in beams. Beams with WGP + SF or DK + SF exhibit the best load-deflection behavior, with delayed crack initiation and improved ductility.
- The calibrated Concrete Damaged Plasticity (CDP) model in ABAQUS demonstrated high accuracy in predicting the nonlinear behavior of AASC. The model effectively captures the transition from elastic behavior to plastic damage, providing a reliable tool for the structural design and analysis of alkali-activated elements without the need for exhaustive physical testing.

### Limitations and future work

While the present study provides valuable insight into the mechanical behavior and flexural performance of AASC incorporating WGP, DK, and SF, its scope is limited to material-level and small-scale testing. The promising performance observed for mixes containing DK and SF highlights the need for follow-up studies on large-scale structural elements, such as full-size beams or columns, to verify the applicability of these results at the structural level. In addition, the long-term durability of these optimized mixes should be investigated under relevant environmental exposures, including sulfate and chloride attack, elevated temperatures, and wet–dry or freeze–thaw cycles, to assess their performance over time. Further research may also examine the response of DK–SF mixes to cyclic, impact, or fire loading, as well as explore alternative fiber types or hybrid fiber systems to further enhance ductility and durability. These targeted studies would help bridge the gap between laboratory-scale results and practical structural applications of AASC.

## Data Availability

The datasets generated and/or analyzed during the current study are available from the corresponding author on reasonable request.
